# Topological properties accurately predict cell division events and organization of shoot apical meristem in *Arabidopsis thaliana*

**DOI:** 10.1242/dev.201024

**Published:** 2022-08-16

**Authors:** Timon W. Matz, Yang Wang, Ritika Kulshreshtha, Arun Sampathkumar, Zoran Nikoloski

**Affiliations:** 1 Bioinformatics, Institute of Biochemistry and Biology, University of Potsdam, 14476 Potsdam, Germany; 2 Systems Biology and Mathematical Modelling, Max Planck Institute of Molecular Plant Physiology, 14476 Potsdam, Germany; 3 Plant Cell Biology and Microscopy, Max Planck Institute of Molecular Plant Physiology, 14476 Potsdam, Germany

**Keywords:** *Arabidopsis thaliana*, Cell division, Classification models, Networks, Shoot apical meristem, Topology

## Abstract

Cell division and the resulting changes to the cell organization affect the shape and functionality of all tissues. Thus, understanding the determinants of the tissue-wide changes imposed by cell division is a key question in developmental biology. Here, we use a network representation of live cell imaging data from shoot apical meristems (SAMs) in *Arabidopsis thaliana* to predict cell division events and their consequences at the tissue level. We show that a support vector machine classifier based on the SAM network properties is predictive of cell division events, with test accuracy of 76%, which matches that based on cell size alone. Furthermore, we demonstrate that the combination of topological and biological properties, including cell size, perimeter, distance and shared cell wall between cells, can further boost the prediction accuracy of resulting changes in topology triggered by cell division. Using our classifiers, we demonstrate the importance of microtubule-mediated cell-to-cell growth coordination in influencing tissue-level topology. Together, the results from our network-based analysis demonstrate a feedback mechanism between tissue topology and cell division in *A. thaliana* SAMs.

## Abstract (precis)

**Summary:** A network representation of live cell imaging data from shoot apical meristems in *Arabidopsis thaliana* is used to predict cell division events and their consequences at a tissue level.

## INTRODUCTION

The adjacency of cells, which species the tissue topology, defines the organization of cells and affects function of organs in multicellular organisms. Therefore, deciphering the organizational principles of cellular connectivity networks are fundamental to improve our understanding of the development of multicellular organisms. The shoot apical meristem (SAM) of plants is a highly organized structure composed of continuously proliferating cells that differentiate and give rise to all aerial organs and is under the control of an intricate signaling network influencing plant growth and response to different stimuli. The SAM epidermis in plants serves as an excellent system to identify organizational principles of cellular connectivity networks (Varner and Lin, 1989).

As the cells in the SAM are glued to each other by a rigid cell wall, changes in the SAM topology, abstracting the tissue with the respective cellular neighborhoods, are only brought about by cell division events. Cell division in plants is a cell size-dependent, cell-autonomous process (Jones et al., 2017), and crossing multiple checkpoints allows the final transition towards cell division (De Veylder et al., 2007; Qi and Zhang, 2020). Willis et al. (2016) recently showed that initial cell size at birth influences the increase in cell size (the sizer model – smaller cells grow faster and all cells divide at a specific size threshold), even though there seems to also be a component of constant size increase (the adder model – always adding the same size increment regardless of the initial cell size at birth) in the SAM of *Arabidopsis thaliana*. This study has hinted at the possibility that a combination of both models may best describe cell division (see D'Ario and Sablowski, 2019 for a comparison of models). Although size-dependent cell division seems to be independent of position and cell-to-cell contact (Willis et al., 2016), a recent study by Jackson et al. (2019) indicates that dividing cells display higher centralities, measured by their participation in more shortest paths between pairs of cells in the network representation of the *A. thaliana* SAM; however, this observation is not sufficient to accurately predict cell division from topological or cell size properties, and is only based on 32 dividing cells from the three upper layers with only seven derived from the surface layer (Jackson et al., 2019).

As biochemical and physical signals are transmitted across tissues and affect cell division, growth and morphology in a spatio-temporal fashion, the question arises of how tissue topology could influence such processes to help the plant respond to a variety of stimuli. In the context of physical signals, the ability of plant cells to respond to growth-driven mechanical signals requires the activity of the microtubule-severing protein KATANIN (Uyttewaal et al., 2012). It has been shown that the lack of mechanical feedback, as seen in the *katanin1-2* mutant, results in changes to the topological features as a consequence of modified cell shape (Jackson et al., 2019). Therefore, this mutant can be employed to test whether topological features are indeed relevant for cell division and related processes.

This issue can be readily addressed due to the availability of plant lines expressing stable fluorescence reporters that allow the monitoring of cellular outlines in combination with confocal imaging techniques (Reddy et al., 2004). In addition, the combination of user-friendly tools for accurate segmentation, such as MorphoGraphX (Barbier de Reuille et al., 2015), with different machine learning (Bhavsar and Panchal, 2012; Pisner and Schnyer, 2020) and deep learning techniques (Camacho et al., 2018) has led to massive advances in the analysis of high-throughput imaging data. Furthermore, the analysis of micrographs for subcellular and cellular scale phenotypes has been facilitated by adopting the network paradigm by abstracting a biological system as a network (Breuer et al., 2017; Nowak et al., 2021). For example, actin filaments can be represented as a network of intersecting paths (Breuer et al., 2017) or the shape of cell membrane can be represented by nodes connected via edges if they do not intersect the membrane (Nowak et al., 2021).

In the context of cellular connectivity network (i.e. topology), nodes correspond to cells and edges represent adjacency of cells. Network properties (topological features) have been employed to study and devise models of cell wall placement for dividing cells, by using the degree (i.e. number of neighbors) in combination with a spring-based model (Gibson et al., 2011) or other individual topological features (Jackson et al., 2019). It has been shown that some of these individual topological features can better predict the placement of some cell walls when compared with a more traditional approach (Jackson et al., 2019), such as dividing cells using the shortest wall placement or generalized Errera's rule (Besson and Dumais, 2011). These resemble the minimization of tensile stress in the central region of the SAM (Louveaux et al., 2016). Although these models provide an important step towards resolving the problem of cell wall placement, each model underperforms on some cells in the central region of the SAM (Shapiro et al., 2015; Jackson et al., 2019).

Furthermore, although individual features have been used in the context of the tissue topology, the whole landscape of topological properties is underexplored. Topological properties can be divided into three groups:

(1) Local properties pertain to individual nodes or edges and can be determined by having access to the immediate neighborhoods. Examples of local topological properties include weighted degree (averaging the edge weights to all neighbors of a given node) or the clustering coefficient, which quantifies the density of connections in the immediate neighborhood of a node.

(2) Global properties pertain to the entire network (e.g. representation of tissue topology) and require knowledge of the entire network to be quantified; e.g. the diameter and the characteristic path length are two global network properties associated with the maximum and average hops for a signal to be transferred from one to another cell.

(3) Local-global properties pertain to individual nodes or edges, but require knowledge of the entire network to be quantified. These include properties related to random walks and shortest paths between two nodes (Table S1).

Although there have been attempts of combining network properties with imaging data from SAMs, there is little progress in predicting individual cells divisions in this plant tissue. Here, we provide a network-based perspective to model cell division and cell wall placement in the SAM of *Arabidopsis thaliana*, a well-established system for studying cell division. To this end, we combine network-based analysis of live cell imaging data with classifiers that allow us to simulate tissue-wise topological changes of the *A. thaliana* SAMs and test these classifiers independently on the *katanin1-2* mutant.

## RESULTS

### Topology and surface area accurately predict cell division events

**Fig. 1.**
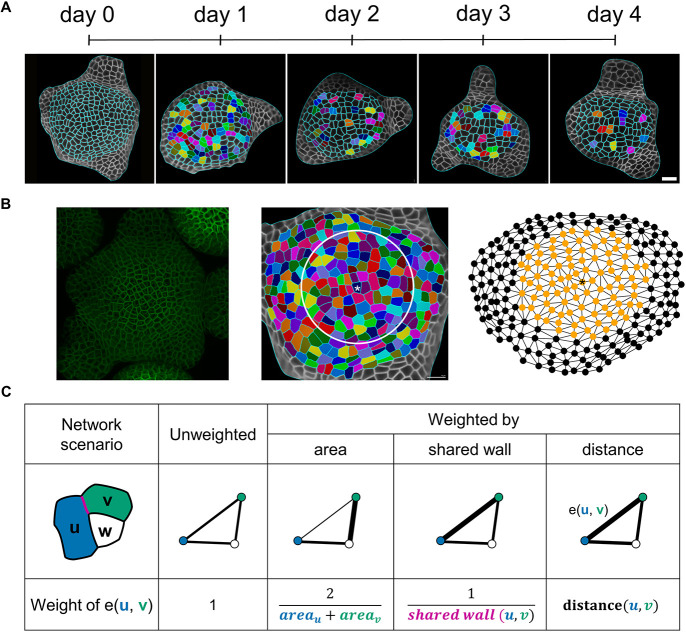
**Feature generation from three-dimensional (3D) images of the shoot apical meristem (SAM).** (A) The surface of *A. thaliana* SAM was imaged every 24 h over 5 days. Pairs of dividing cells, depicted with the same colors, were determined manually (see Materials and Methods). (B) The 3D images of SAMs were converted to 2.5D surfaces by employing MGX (Barbier de Reuille et al., 2015) (left panel). The surface is abstracted according to its topology, capturing the connectivity of neighboring cells in a radius of 30 μm (white circle) around the central cell(s), marked with an asterisk (center panel). The topology of the analyzed cells inside the circle is colored orange (right panel). Two nodes are connected by an edge if the cells they represent share a cell wall. (C) Four different network scenarios are considered: (1) unweighted edges, and edges weighted by (2) area, (3) a shared cell wall and (4) distance, illustrated for the case of three cells u (blue), v (green) and w (white). In the unweighted network scenario, all edge weights have a value of one. The edge weight for the network weighted on area, shared wall and distance is the inverse of the mean cell areas of u and v, the inverse of the shared cell wall area (magenta) and the inverse distance of the center of mass for the graph weighted on the distance (black). The weights of the edge e(u, v) in the four scenarios are illustrated with different line widths (a smaller thickness represents smaller weight values and cells being closer in terms of topological representation).

The question of whether division of a cell embedded in a tissue is driven by the topology of the neighboring cells, the area of the cell, or combination of the two is still unanswered. To address this question, we imaged eight SAMs of *A. thaliana* expressing a plasma membrane reporter (pUBQ10:acyl-YFP) every 24 h over 5 days using confocal microscopy (Fig. 1A). First, we manually determined the number of dividing and non-dividing cells between two consecutive time points in the central zone of the SAM. We defined the central zone of a SAM as the area covered by a circle of 30 µm radius around the most central and highest point in the analyzed SAM (Fig. 1B), and found that 28.9±17.9% of cells divided per tissue between two successive tissue time points, with a total number of 605 dividing cells and 1458 non-dividing cells in 28 tissues (Fig. 1B).

Next, we represented the topology of the full tissue as a network, in which every node corresponds to a cell and two nodes are connected by an edge if the cells share cell wall. As a result, the topology abstracts the tissue from an image into a neighborhood representation. For each cell in the central region, we calculated 17 properties, referred to as topological features (Table S1), in an unweighted network, in which every edge is of weight 1. We also applied different edge weights, which add cellular information into the topology based on the mean outer periclinal surface area, shared cell wall and distance of the cell centroids between two nodes representing those cells (Fig. 1C). In addition, we considered the surface area of each cell in the central zone as a biological feature for division prediction.

Previous studies have shown that a critical cell size threshold exists for cell division in the SAM of *A. thaliana* (Jones et al., 2017). To show that topological features capture information distinct from that provided by the cell surface area, we calculated its Pearson correlation coefficient (*r*) with the topological features (Fig. S2). We found that the degree of distance weighting was most closely correlated to the surface area (*r*=0.75). Nevertheless, the absolute value of the correlation with surface area was smaller than 0.3 for 47% of the features (98% showed correlation less than 0.7), with only three of the 50 considered features showing non-significant correlation (*P*<0.05, Table S2). Therefore, topological features in the considered network scenarios carry information that is different from that obtained by the cell surface area alone. To further show the predictive power of the classifiers trained on the topological features, we considered two reduced feature sets that included only features with an absolute value of the Pearson correlation coefficient (*r*) smaller than 0.3 (Fig. S2). In this way, we aimed to remove bias by considering features that may, to a certain extent, include information about surface area. As a result of these considerations, we trained five classifiers based on support vector machines (SVMs) with linear kernel (Bhavsar and Panchal, 2012) to predict cell division based on: all topological features (topo); surface area alone; the combination of topological features and surface area (topo+area); topological features with low absolute value of correlation with the surface area (*r*<0.3); and unweighted topological features. To this end, we selected all dividing and non-dividing cells from 20 tissue time steps (derived from six SAMs) for training the SVMs, and ensured balanced performance by weighting based on the frequency of dividing and non-dividing cells. We kept the data from the remaining eight tissue time steps (two SAMs) as a testing set (see Materials and Methods). Furthermore, we partitioned the 1445 selected cells into training and validation sets, and used six-fold cross validation to train the classifiers with each split being on plant (see Materials and Methods).

**Fig. 2.**
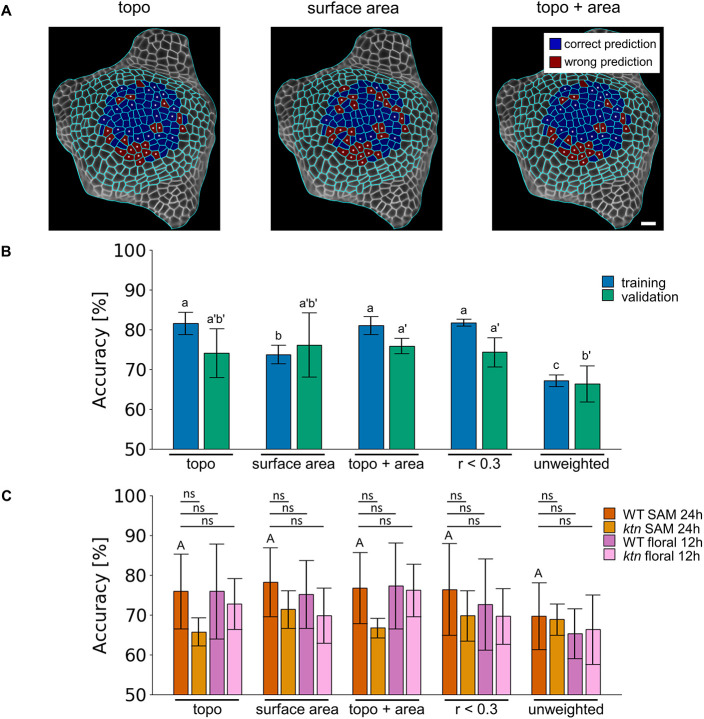
**Surface area and topology-based features generate similar predictions of cell division in the SAM that generalize for *ktn* mutant and floral meristems central region.** (A) Comparison of predicted and observed division events on wild-type shoot apical meristem (SAM) test plant tissue from Fig. 1A day 0, highlighting correct predictions in blue and wrong predictions in red. Dividing cells are indicated with white dots. The predictions are from classifiers trained on different feature sets: combined topological features (topo), surface area and combined topological features with surface area (topo+area; from left to right). Scale bar: 10 µm. (B,C) The accuracy of the support vector machine classifier to predict cell division of (B) wild-type SAM training (blue) and validation (green) in 24 h time steps, and (C) testing wild-type (dark orange) and *ktn* mutant (dark purple) SAMs in 24 h time steps, and wild-type (light orange) and *ktn* (light purple) floral meristem in 12 h time steps. Datasets from left to right: topo, surface area, topo+area, a reduced set of topological features that show an absolute Pearson correlation coefficient with surface area smaller than 0.3 (*r*<0.3), and unweighted topological features (unweighted; derived from the unweighted network scenario). The performance on the training and validation set is determined from sixfold cross-validation as mean±s.d. The 10 most important features of the test classifier trained on the feature set *r*<0.3 (coloring positive blue and negative coefficients orange) are shown. Different letters indicate significance between groups using Benjamini-Hochberg adjusted two-tailed paired *t*-test (*P*<0.05). Statistical testing for differences of classifier performance for the training, validation and test sets is conducted separately (lowercase letters without prime, lowercase letters with prime and uppercase A, respectively). Lines indicate a Benjamini-Hochberg adjusted two-tailed Student's *t*-test between test wild-type SAM and other central apical meristems with non-significant (ns) pairs. *N*_WT_=28 (20 tissues time steps for training-validation and eight for testing); *N_ktn_*=5; *N_WT_floral_*=8; *N_ktn_floral_*=7; *n*_WT_=1445 and 618 train-validation and test cells, respectively; *n_ktn_*=575; *n_WT_floral_*=151; *n_ktn_floral_*=295. *N* denotes the number of tissues over the time steps considered; *n* denotes the number of train-validation cells.

Although the training accuracy of the SVM using only the surface area was 73.8%, the training accuracy solely based on topological features was significantly higher – 81.6% (*P*=0.0094, paired *t*-test); this was also the case when the combined set of topological features and surface area was used, with a training accuracy of 81.2% (*P*=0.0070, paired *t*-test) showing that all feature sets contained information to be learned in order to predict cell division events. To ensure no overfitting, i.e. the model memorizing only training data, and to ensure transferability on unseen data, we applied the model to validation and test data. Here, we observed a similar performance, with no difference in the validation accuracies between the feature sets for the three SVMs (∼75%; see Fig. 2B). For the test SAM, the classifier based on the surface area alone exhibited the best performance, with an accuracy of 78.3%, closely followed by the SVM that was trained on the combination of topological features and area (76.7%), and the SVM that considered all topological features (75.9%, see Fig. 2C, Table S3). The area under the curve (AUC) of the receiver operating characteristic (ROC) curve, which is used as another measure of performance, yielded similar results (Fig. S3A, Table S3).

The removal of topological features that were highly correlated with area does not change the performance for training or for validation and test accuracy (Fig. 2B,C, Table S3). Although using only the topological features from the unweighted network scenario (Fig. 1C) decreased the training performance, the validation accuracy was only significantly reduced compared with the classifiers trained with both topological features and area, and when *r* < 0.3 (*P*=0.0144 and 0.0300; paired *t*-test). The final test accuracy was slightly reduced, but was comparable with that of the other classifiers at 69.8% (Fig. 2B,C, Table S3). Inspection of the learning curves showed that the classifiers did not suffer from high bias and variance, and that the training set was sufficiently large (Fig. S4). Therefore, we concluded that topological features and area can predict cell division independently of each other, whereas the performance could not be further increased by the combination of both feature types.

We hypothesize that feedback between topology and cell division is mediated by cell-to-cell interactions, suggesting that properties related to signal transduction and their influence in the network are important features in classification. Indeed, we found that harmonic centrality, which measures the sum of reciprocal distances of a node to all others, is of highest positive importance (Fig. 2D). Harmonic centrality implies that nodes that are closer to all nodes (Marchiori and Latora, 2000), and hence in the center of topology, are more likely to divide. Similarly, the next two positive most important features are based on Katz centrality. This centrality measure depends on the number of walks, rather than paths, of a particular length, whereby nodes that are involved in more walks to the others are considered more central (Katz, 1953), and has a similar interpretation to harmonic centrality. Interestingly, and in line with the previously discussed features, we also found that the versions of information centrality that weight the connections of cells (edges) based on shared wall, on equality (unweighted) and on distance are also of high importance in classification. If this centrality index measures the harmonic mean length of paths ending at a given node, suggesting the node is connected via many short paths to all other nodes, then it will be smaller. In addition, weighted node degree, absolute graph density in the second neighborhood and clustering coefficient denote the local density of subgraphs induced by a given node, suggesting that topologically dense regions are hotspots of cell divisions.

To further corroborate the biological relevance of these findings, we randomly permuted the labels and retrained the classifiers, repeating this procedure 100 times for each feature set (see Materials and Methods). We could not train a classifier that performed well and was able to generalize on the validation or test set, and exhibited accuracies expected by chance (Fig. S5A). Therefore, the classifiers trained on the randomized labels demonstrate that the features used capture information that is important for classification of dividing and non-dividing cells at 1-day intervals.

To evaluate the transferability of the classifiers, we applied the SVMs trained on wild-type SAM data on the same feature sets generated from three different central meristems: SAMs of the *katanin1-2* (*ktn*) mutant, and floral meristems of wild type and *ktn* (tracked every 12 h because of their increased division speed compared with the SAM). Although the performance for predicting cell division events on the floral meristems was at the same level as the wild-type SAM test performance, the performance of the classifier slightly decreased in all feature sets from the *ktn* SAMs (non-significantly for accuracy and significantly reduced for area under the curve of all classifiers), except for the unweighted topological features (Fig. 2C, Fig. S3B). Although these findings demonstrate the importance of surface area as a determinant of cell division, they also support the claim that topology plays an important role in predicting cell division events and that similar mechanisms exist in the central regions of both SAM and floral meristems.

### Combination of topological and biological features enables recreation of the local topology after cell division

To examine whether properties derived from the tissue connectivity network as well as biological properties (i.e. cell size and perimeter, distance, and shared cell wall between cells) are informative in the time-dependent connectivity of daughter cells, we trained classifiers based on SVMs (with linear kernel) to predict which of the cells adjacent to a dividing (parent) cell are neighbors of the divided (daughter) cells. We distinguished neighbors that were adjacent to only one daughter cell: those adjacent to the daughter cell closer to the SAM center are labeled as class 0; those adjacent to the daughter further from the center are classified as class 1; neighboring cells adjacent to both daughter cells are considered to be class 2 (Fig. S1C).

To predict changes in the local topology due to cell divisions, we first determined all neighbor-parent pairs of dividing parental cells and then predicted the adjacency of the neighbor to the daughter cells. To this end, we considered the topological features as well as biological properties of the parent and the neighbor cells in the preceding time point. We used the topological features of the neighbor cell to distinguish neighbor-parent pairs in which a neighbor is adjacent to two dividing cells. By including both parent and neighbor, we generated 96 unique topological-based properties for each neighbor-parent pair of the combined topological feature set (topo, Fig. S1B). For the biological feature set, we extracted the surface area and perimeter of both the parent cell and neighbors, as well as their shared cell wall and distance between the centers of mass. For the combined features, we concatenated both topological and biological features. Given a parent cell, we determined the class of its neighbors at the next time point by aligning the tissues at 1-day time intervals manually, and determining their adjacency with their neighbor with respect to the daughter cells (see Materials and Methods and Fig. S1B).

**Fig. 3.**
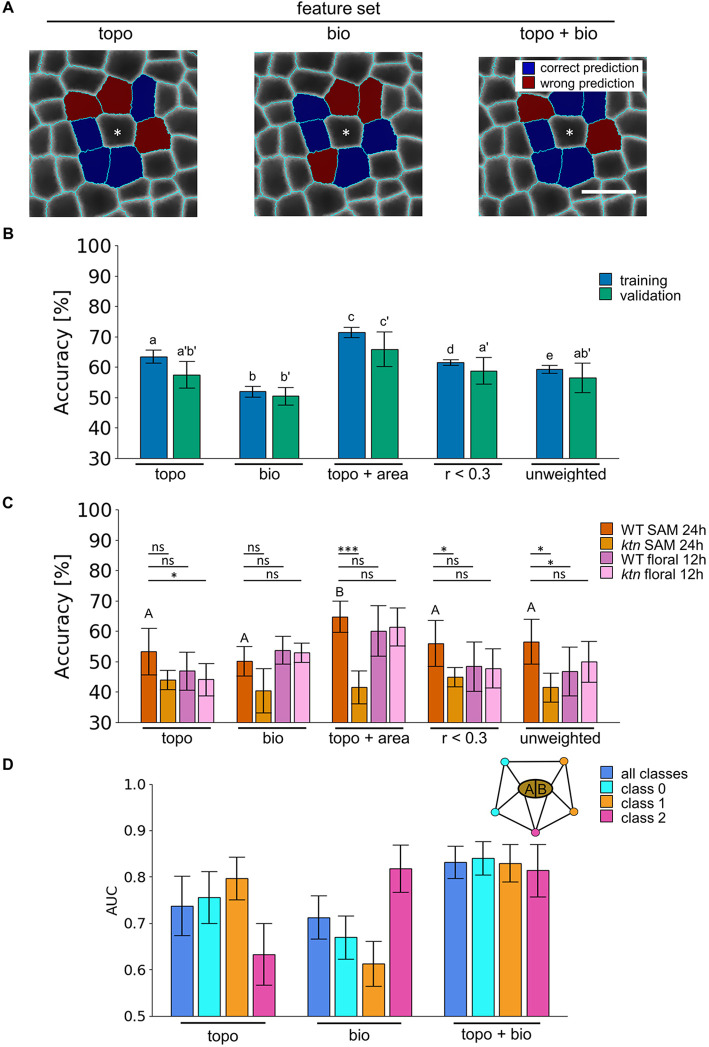
**Topological and biological features are required for accurate prediction of the local neighborhood after cell division in the SAM and *ktn* mutant shows reduced performance on SAM.** (A) Comparison of predicted and observed local neighborhood changes around dividing cell (white asterisks) from wild-type shoot apical meristems (SAM) tissue after 24 h (as in Fig. 1A, day 0), highlighting correct (blue) and wrong (red) predictions. The predictions are made with classifiers trained on different feature sets: combined topological (topo, including features of the four network scenarios, see Fig. 1C, Fig. S1), biological (bio, including area, perimeter, shared cell wall and distance), and combined topological and biological features (topo+bio). Scale bar: 10 µm. (B,C) Accuracy of the support vector machine classifier to predict local topological changes for dividing cell neighbor pairs of (B) wild-type SAM training (blue) and validation (green) in 24 h time steps, and (C) testing wild-type (dark orange) and *ktn* mutant (dark purple) SAMs in 24 h time steps, and wild-type (light orange) and *ktn* (light purple) floral meristem in 12 h time steps. (D) Area under the curve (AUC) of the receiver operating characteristic based on the wild-type SAM test data for the local neighborhood prediction of all classes (blue), and for the class of neighbors adjacent to the daughter cell (cell A, see legend in B) closer to the SAM center (denoted as class 0; cyan), adjacent to the daughter (cell B, see legend in C) farther from the center (denoted as class 1; orange) or adjacent to both cells (denoted as class 2; magenta). The classifiers are trained on topo, bio, topo+bio, reduced set of topological features that show an absolute Pearson correlation coefficient with all biological features smaller than 0.3 (*r*<0.3), and unweighted topological features (unweighted; derived from the unweighted network scenario). The performance on the training and validation set is determined from sixfold cross-validation as mean±s.d. Different letters indicate significance between groups using Benjamini-Hochberg adjusted two-tailed paired *t*-test: *P*<0.05. Statistical testing for differences of classifier performance for the training, validation and test sets was conducted separately (lowercase letters without prime, lowercase letters with prime and uppercase letters, respectively). Lines indicate a Benjamini-Hochberg adjusted two-tailed Student's *t*-test between test wild type and other central apical meristems. ns, non-significant, *P<0.05, ***P<0.001. *N*_WT_=28 (20 tissue time steps for training-validation and eight for testing); N*_ktn_*=5; N*_WT_floral_*=8; N*_ktn_floral_*=7; *n*_WT_=2103 and 912 train-validation and test cells, respectively; n*_ktn_*=1042; n*_WT_floral_*=1646; n*_ktn_floral_*=2003.

We excluded neighbor-parent pairs for which the neighbor also divided in the 1-day interval being considered to avoid bias from guessing which cell divides first (Fig. 3A). Using this procedure, we created 3015 neighbor-parent pairs with 900 representatives in each of the three classes (0, 1 and 2) from eight different SAMs, tracked every 24 h over 5 days. The data were split into three parts (training, validation and test data), such that the SAMs of two plants were kept as test data, while the rest of the plants were used in a nested fivefold cross-validation for training the SVM.

The training and validation accuracy was best for the SVM based on the topological features combined with biological features, at 71.6% and 65.9%, respectively. The topological and biological features alone showed 12.8% and 23.4% reduction in validation accuracy compared with the combined classifier, and similar reduction in training accuracies (*P*=0.012, paired *t*-test with Benjamini-Hochberg correction). Regarding the performance on the test set, the combined classifier performed best, with an accuracy of 64.8%, followed by the classifier based on topological features alone with 53.3%. The classifier that used biological features alone had the worst accuracy (equivalent to guessing) of 50.2% (Fig. 3B, Table S4).

Investigating the area under the ROC curve (AUC) measure for individual classes highlighted the differences between the two classifiers trained on topological or biological features alone. The SVM based on the topological features showed better performance for the neighbors adjacent to only one cell (class 0 and 1) in comparison with the classifier based on the biological features (i.e. relative increase of 13.0% and 30.0% for class 0 and 1, respectively). In contrast, the SVM based on the biological features performed 29.4% better for neighbors adjacent to both daughter cells in comparison with the classifier based on the topological features. Combining both feature sets improved the average AUC on the validation data of the classifier by 12.7% and 16.7% (relative increase compared with topological and biological features alone, respectively) while retaining high performance for all classes (Fig. 3C, Fig. S6). Investigating the reduced topological feature set (i.e. removing features with Pearson correlation coefficients larger than 0.3 with any biological feature) as well as considering only unweighted features resulted in similar validation and test accuracies compared with all topological feature trained classifiers (Fig. 3B,C, Table S4). Investigating the classifier learning curves reveals that they did not suffer from high bias and variance, and that training was carried out on a sufficiently large number of pairs (Fig. S7). These findings indicate the importance of both topological as well as biological properties in predicting local topology after a division event.

To further corroborate the biological relevance of these results, we randomly permuted the labels and retrained the classifiers, repeating this procedure 100 times for each feature set. Although the resulting classifiers showed performance better than expected at random on the training data with the three sets of features, they did not generalize well and exhibited accuracy on the validation set similar to that expected by chance (Fig. S5B). Furthermore, we investigated the more difficult scenario of including neighbor-parent pairs whose neighbors also divide and repeat the topology prediction procedure. Here, we found similar performance to that on the training, validation and test sets for all combinations of feature sets (Table S4). Therefore, our findings demonstrated that the used features capture information important for classifying changes in local topology predictions surrounding dividing cells in 24 h intervals.

We also tested how well the trained classifiers based on the wild-type SAM data performed on the *ktn* mutant SAM and floral meristem, as well as on wild-type floral meristem. With the data from the *ktn* mutant SAM, we found a reduction in accuracy for the best-performing classifier when trained on the combined topological and biological, reduced feature set, and unweighted features (35.7%, 19.9% and 26.6% with *P*<0.001, *P*<0.0463 and *P*<0.0103 using Student's *t*-test, respectively, Fig. 3C). We could equally well predict floral meristem topological changes compared with the wild-type SAM test data, except for *ktn* floral meristem based on all topological features and wild-type floral meristems based on unweighted topological features (with relative reduction of 17.2% and 17.4%, and *P*=0.0462 and *P*=0.0490 using Student's *t*-test, respectively). The reduced performance on SAM data of classifiers trained on topological and biological features combined cannot be attributed to one specific class (Fig. S1C). These results highlight the importance of both topological and biological information in local topology rearrangement after cell division.

### Combined application of division event and local topology prediction enables the prediction of tissue topologies

**Fig. 4.**
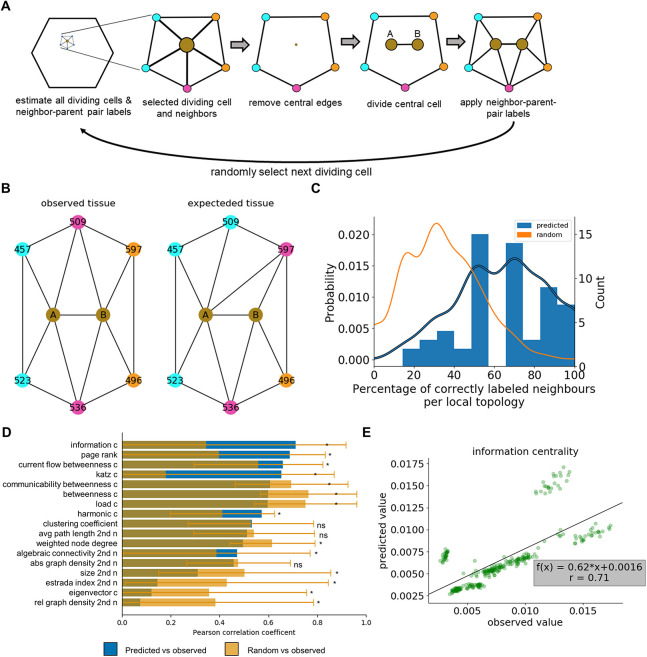
**Concordance between observed and predicted topologies.** The cell connectivity network of the two test plants was predicted by applying classifiers for division event and topology prediction. (A) Illustration of the procedure applying the division and local topology classifiers to generate the topology of the next time point (24 h time interval). After division and topology prediction, a dividing cell (brown dot ) is selected along with its neighbors (blue circles) and its adjacency relationship (edges, black lines) (left schematic). The selected cell (predicted to divide) along with the edges incident to the corresponding node are removed and replaced by the divided daughter cells (‘A’ and ‘B’; representing the cell closer and farther away from the SAM center) that are adjacent to each other (middle three schematics). The daughter cells are connected with their neighbors based on the prediction from the local topology classifier (right schematic). The next dividing cell is randomly selected and the previous steps are repeated until all dividing cells are selected. (B) One example of the predicted local topology with an overall accuracy of 66.6% for the full local topology is compared with the observed local topology. The divided daughter cells ‘A’ and ‘B’ (brown) are adjacent to the predicted or observed cells (numbers indicate the same cells) coloring their respective parent-neighbor class (cyan or orange indicate a cell connected only with daughter ‘A’ or ‘B’, respectively; magenta indicates a cell adjacent to both daughter cells). (C) Histogram and density plot of the percentage of correctly estimated neighbors per local topology of cells predicted as dividing in the test plants (blue) are compared with the density plot of randomly assigned parent-neighbor classes (orange). The difference between distributions is tested using a Kolmogorov–Smirnov test, *P*<0.001. *N*=8 tissue time steps, *n*=59 local topologies. (D) The concordance between the observed and predicted topologies was quantified (mean, blue) for non-dividing cells in both topologies by calculating and ranking the Pearson correlation coefficient based on 17 topological features from the unweighted networks (see Fig. 1C; Table S1). The procedure was repeated dividing all cells predicted to be non-dividing, randomly assigning classes to the neighbors and calculating the correlation as described before (data are mean±s.d, yellow). Benjamini-Hochberg-corrected two-tailed one-sample *t*-test: *P<0.05; ns, non-significant. (E) The observed information centrality is plotted against the predicted information centrality for all non-dividing cells and the best linear fit (solid line) with its function f(x) and the respective Pearson correlation coefficient (*r*) is overlaid. *N*_WT_=8 tissue time steps, *n*_WT_=290 cells not dividing in both observed and predicted tissue.

To apply the classifiers and compare the resulting topologies, we used the data from the test plant and successively predicted division events and changes in local topology using classifiers trained on the combined biological and topological features (see following procedure outlined in Fig. 4A). We compared the predicted and observed topologies by investigating the unweighted topological features (Fig. 1C, Table S1) of non-dividing cells in the next time points of both scenarios. We selected non-dividing cells of both scenarios, i.e. predicted and observed, to compare in a pairwise manner their unweighted topological features. We did not consider other network scenarios (see Fig. 1C) as we would need to estimate the weights for the topology, adding a layer of uncertainty. Here, 290 of the possible 437 non-dividing cells in the observed topology were also non-dividing in the predicted topology. For these cells, we calculated *r* of all unweighted features between observed and predicted topologies, with the information centrality showing the largest value of *r*=0.71, and 10 out of 17 features with *r*>0.5 (Fig. 4D, blue bars). We compared the predicted and observed values of harmonic centrality of the non-dividing cells of the next time step, and found strong correlation (Fig. 4E, Fig. S8).

For comparison, given the same test plant observed topologies, we selected all non-dividing cells predicted to divide and randomly connected the neighbors with the divided cells, which reoriented the local neighborhood; we then repeated the correlation analysis of the resulting topology with the observed topology. This ‘random propagation’ scenario allowed us to construct and investigate the most opposite example to our predictions (Fig. 4D, orange bars). Comparing the predicted and random propagation correlations showed that six out of 17 topological features had a higher correlations and eight had a lower correlation (one sample *t*-test with Benjamini-Hochberg correction, *P*<0.05). Although local features (calculated on the second neighborhood, i.e. the topology induced by cells in a radius of two cells around a specific cell), such as size (number of neighborhoods) and relative graph density (average number of neighbors per cell), showed small correlations, suggesting a need for a correction in the case of erroneous topology recreations (e.g. creating non-planar topology), the overall structure in terms of flow centralities showed more and stronger correlations for the predicted propagation (Fig. 4D, blue versus orange bars).

To further investigate the performance of the local topology prediction on the test plant, we calculated the percentage of correctly predicted neighbors for each cell dividing in the predicted and observed tissue (example in Fig. 4B). The distribution of correctly labeled neighbors per dividing cell was significantly shifted towards higher accuracy when comparing the predicted and random topology (Fig. 4C).

## DISCUSSION

The biochemical pathway of cell division control has been extensively studied (Dewitte and Murray, 2003), but only recently have external cues also been considered in order to understand the effect of cell divisions in tissues (Hartig and Beck, 2006; Shimotohno et al., 2021). It is known that the outer epidermal cell wall resists most forces (Beauzamy et al., 2015), and, thus, division in the SAM outer layer needs to serve a meristematic function and provide mechanical protection. This raises the issues of whether cell division and the subsequent local topology rearrangement are affected by tissue topology and whether tissue topology contains sufficient information for its accurate prediction.

Based on our extensive network-based modeling, we showed that both surface area, as an approximation of cell size, as well as the characteristics of topology, allow for prediction of cell division events in the central epidermal region of *A. thaliana* SAM, in contrast to earlier reports showing only an increase in size between dividing and non-dividing cells (Jackson et al., 2019). With the local topology rearrangement predicting model, we provide an alternative and network-based model to infer the changes applied to the network due to cell divisions. In addition, we successfully applied the division and local rearrangement classifier trained on wild-type SAM to the central region of the wild-type floral meristem, suggesting a similar mode of action. At the molecular scale cyclin-dependent kinase (CDK) G1 is known to bind DNA and serves as a ruler after cell division, allowing for size-dependent division in *C. reinhardtii* (Li et al., 2016), whereas KIP-related protein 4 has a similar function in the *A. thaliana* SAM niche (D'Ario et al., 2021). Modeling cell division in the SAM of *A. thaliana* also revealed the importance of CDKs in G1-S and G2-M phase transition (Jones et al., 2017). Furthermore, the work of Willis et al. (2016) showed that cell division events in SAMs of *Arabidopsis* treated with naphthylphthalamic acid, an inhibitor of auxin transport that generates naked meristem, are influenced by both cell size increase and a cell size threshold. Both models explain the importance of surface area in predicting cell division events, but they do not explain the importance of topological features. Here, the mechanical feedback loop, manifested by the ability of cells to react to changes in turgor pressure produced by microtubules and cell wall rearrangement affecting cell divisions (for details of the feedback loop, see Sampathkumar, 2020), could explain the link between topology and the summed turgor and supracellular mechanical stress. Alternatively, the predictive ability of topological properties may result from long-distance communication of different phytohormones (Shimotohno et al., 2021) or could be due to cell-to-cell communication by plasmodesmata (Kitagawa and Jackson, 2017).

However, both cell division and the cell wall positioning affect tissue organization; a prime example is the effect of division patterning in lateral root initiation (von Wangenheim et al., 2016). Our study relies on the adjacency of cells in the tissue topology, in contrast to other cell wall models, such as the generalized Errera's rule (Besson and Dumais, 2011), the spring-based model (Gibson et al., 2011) and the mechanical stress related model (Louveaux et al., 2016), that predict the placement of the cell wall based on the individual cell geometry. Our classifier employs the biological feature set composed of six cellular features, with limited information about the dividing and neighbor cell geometry, and allows reliable prediction of changes in the local topology. These local changes in the topology mirror the effect of cell wall placement on the tissue. In addition, we show that topological features alone suffice to accurately predict local topological changes. Although single topological properties are already used to estimate cell wall placement, the percentage of dividing epidermal cells in this study was only 5.2% (total *n*=32/582 dividing and non-dividing cells) per tissue every 11 h (Jackson et al., 2019). In contrast, our results rely on experiments in which cells divided more regularly, with an average of 28.9% of dividing cells per tissue every 24 h (total *n*=605/1458 dividing and non-dividing cells), allowing us to train robust classifiers. We show that the combination of both feature sets boosts performance of local topology reorientation prediction (Fig. 3), indicating that the inclusion of multiple viewpoints of information available to cells needs to be involved to solve the problem of cell wall placement in the SAM. This raises the question of how information of the topology is biologically transferred over the tissue, either via mechanical or via active and passive transduction of biochemical signals.

To demonstrate the generalizability of the classifiers, we show that they can be used to make accurate predictions for *ktn* mutants that are defective in mechanical feedback regulation. Our results indicate similar performance for the classifiers with biological feature sets from wild type and *ktn*. In contrast, the classifiers trained on topological features show reductions in performance in *ktn* compared with wild type. This difference in performance is not due to differences in topological features, as the normalized features showed similar distributions (Fig. S9). These results suggest a potential role for KATANIN in linking sub- and supracellular mechanical stress, which are known to affect leaf epidermal cells (Eng et al., 2021), and a role in positioning of the preprophase band, spindle and phragmoplast (Komis et al., 2017). In addition, the cell geometry of the *ktn* mutant differs from the wild type and might also influence the topology. Therefore, the combination of network-based modeling with machine learning provides a method for screening SAMs under different conditions and mutants. More specifically, reduction in test performance of the classifiers trained either on mutant surface area or on topological features compared with the wild type could suggest effects that disturb only functions related to the cell cycle or to a topological effect.

When combining division prediction and the resulting changes to the tissue, previous studies mostly focused on single cell division or on propagating tissues based on division likelihoods using the number of neighbors (Gibson et al., 2011) or using only area as a fixed threshold (Sahlin and Jönsson, 2010; Alim et al., 2012). Our classifier, on the other hand, incorporates more diverse tissue-level information. Here, we have combined our best classifiers to predict future tissue topology using the combined topological and biological features. Although the results of this propagation of classifiers are promising, the careful inspection of the findings, particularly with respect to planarity and topological properties of the reconstructed topologies indicate that further research should consider simultaneous modeling of cell neighborhoods of higher orders to improve the reconstruction.

Furthermore, as information is not only being passed along the epidermis (L1-layer), the assessment of cell division events and their effects on the topology could be expanded beyond the epidermis of the SAM, as we know that the L2 and L3 layers play a vital role in supporting the meristematic function through the feedback of CLAVATA and WUSCHEL (Schoof et al., 2000). Transferring the classifiers to other plant species, such as maize (which has only two distinct layers forming the SAM), may provide insights into how meristematic function can be conserved with fewer cells. As other tissues and organs are also experiencing mechanical stresses, hormone gradients and other transport related feedbacks, e.g. growth-related mechanical stress (Sampathkumar et al., 2014), auxin gradients (due to PIN; Shi et al., (2018)), soil density in roots and bending through wind in the stem, there are bound to be feedback loops of cells and tissues to sense and react to those cues on a topological level to integrate this information into the plants development.

## MATERIALS AND METHODS

### Plant materials and growth condition

We grew *Arabidopsis thaliana* wild-type (Wassilewskija ecotype) plants with the membrane reporter pUBQ10::acyl-YFP (previously described by Willis et al., 2016) and *katanin1-2* mutant in Columbia-0 background with the membrane reporter Lti6b-GFP (Eng et al., 2021) in short day (8 h/16 h day/night), 20°C/16°C conditions for 3 weeks and then transferred to long day (16 h/8 h day/night), 20°C/16°C conditions until shoot apical meristem sampling. We cultured sampled shoot apical meristems (SAMs) in transparent imaging boxes containing apex culture media under long day, 22°C conditions as previously described (Wang and Sampathkumar, 2020).

### Time-lapse data acquisition and pre-processing

We acquired confocal *z*-stacks (3D images) at an excitation wavelength of 514 nm and 488 nm for imaging YFP and GFP, respectively, with a 40×/0.8 water immersion objective of wild-type SAMs every 24 h for 5 days or *ktn* SAMs every 24 h for 3 days, and floral meristems of wild type and *ktn* every 12 h. Next, we used MorphoGraphX (MGX) (Barbier de Reuille et al., 2015) to obtain a 2.5 D surface mesh of the meristem L1 layer from the 3D images with at least two dividing cells; from there, we extracted the cellular connectivity network (topology). In addition, we measured the shared cell wall of the neighboring cells (MGX function: Mesh/Export/Save Cell Neighborhood 2D), the surface area and cell positions (MGX: Mesh/Heat Map/Heat Map Classic). The cellular connectivity network is composed of nodes, representing the centroids of the extracted cells. Edges connect two nodes if the corresponding cells are adjacent to each other, e.g. share a cell wall. We lineage tracked all cells for 1-day for SAMs and in 12 h time steps for floral meristems manually in MGX, resulting in tissue time steps with distinct topologies (Fig. 1A). We refer to dividing cells, at time t (days), as parent cells, and to their descendants, at time t+1 (days), as daughter cells. To select the cells for the downstream analysis, we first manually determined the cells closest to the center of the SAM surface, approximated by the highest curvature, then determined the largest distance to the boundary regions and emerging primordia. To this end, we compared the positions of cells with the average position over all cells.

### Prediction of dividing cells

To predict cell division events (*n*) of central and non-peripheral cells, we employed python 3.8.1 for all further steps, e.g. to select all cells in a radius of 30 µm around the center for SAMs and 15 µm for floral meristems (Fig. 1B). Accordingly, we analyzed central cells and excluded peripheral cells. We considered a cell as peripheral with respect to a connectivity network when the topology induced by the adjacent nodes did not form a cycle. We then labeled each of the selected cells as a dividing (1) or non-dividing (−1) cell within each time step. In addition, we determined six sets of features (see below; for unreduced sets, see Fig. S1) for each cell.

Four out of five feature sets are based on entire tissue topology (i.e. including peripheral cells as well as cells outside the central region from the cellular connectivity network) and consist of topological features for all central cells; the fifth set includes only the surface area of the central cell. When calculating the topological properties, we considered different scenarios for weighting the edges. In the case of the unweighted topology, we weighted all edges equally (edge weight=1). For the area-induced topology, we used the inverse of the mean surface areas of the two adjacent cells as edge weight. For the wall-induced topology, we defined the edge weights as the inverse of the shared cell wall area between two cells. For the distance-induced topology, we determined the distance between the centroid positions of two adjacent cells as the edge weight (Fig. 1C). We calculated ten topological properties for each central cell and network scenario (see Table S1). Furthermore, we considered topological properties based on the induced subgraph of the first neighborhood (see Table S1). We estimated all properties in python using the networkx 2.4 package.

To train the classifiers for prediction of division events between two successive time points, we split the wild-type data from the eight plants into two datasets, a training-validation and a testing set, with six and two plants, respectively, while keeping three *ktn* plants, and two wild-type and three *ktn* floral meristems as a separate test set (resulting in 20 training-validation and 8 testing wild-type SAM, 5 *ktn* SAM, 8 wild-type and 7 *ktn* floral meristem tissue time steps, *N*). We split the data into training-validation and test sets to ensure no overfitting was carried out and rigorously examined our classifiers. As there are fewer dividing cells, their class is the minority class and we account for the imbalance by weighting the classifier and performance measures accordingly. We applied a support vector machine (SVM) with a linear kernel to predict the occurrence of cell division events within one time step, e.g. 1 day. To this end, we used the five different feature sets, the unweighted topological features (unweighted topology), all topological features combined (topo), the surface area (surface area), topological features and area (topo+area), as well as two reduced feature sets, including only topological features with Pearson correlation coefficients with a surface area smaller than 0.3 (denoted by *r*<0.3) (Fig. S1A). We trained each classifier with the topological properties as features of the training-validation set using six-fold cross-validation and always kept one plant as a validation set.

To this end, we *z*-normalized [(X-mean)/s.d.] the topological properties with the corresponding mean and standard deviation (s.d.) for the train-validation and the wild-type test datasets. The *ktn* data were *z*-normalized using means and standard deviations. We estimated the hyperparameters on the training set by sixfold cross-validation using a grid search (sklearn 0.22.1, model_selection.GridSearchCV) with 100 regularly spaced hyper-parameters for each power of 10 (C: 10^−4^-10^1^). We further tested the classifiers by retraining the SVMs on all training-validation data with newly selected parameters and applied them on the unseen test data. We quantified the performance of the classifiers by calculating five measures of performance, including: the accuracy, F1-score, true positive rate, false-positive rate and area under the curve (AUC) of the receiver-operator characteristic (ROC). For comparative analysis between two performance measures, p1 and p2, we used the relative difference (100 
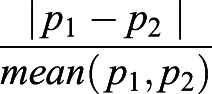
). We quantified the difference between feature sets of training, validation or testing using a paired *t*-test (scipy 1.8.0, stats.ttest_rel) and the difference between testing of wild-type SAM and *ktn* SAM, and wild-type and *ktn* floral meristem using Student's *t*-test (scipy.stats.ttest_ind) with Benjamini-Hochberg testing correction (statsmodels 0.13.2, stats.multitest.multipletests).

To further inspect the training of the classifiers, we generated the learning curves by retraining each classifier on a different number of training data (keeping the hyper-parameters from above). We further determined the feature sets information content by shuffling the labels 100 times, retraining the classifier using the default linear SVM parameters (sklearn 0.22.1, svm.SVC) on each set of shuffled labels, and calculating the performance of the resulting classifiers.

### Recreating of local topology after cell division

For the prediction of the changes in local topology of dividing cells, we automatically selected all non-peripheral neighbor-parent pairs of dividing cells (*n*). Next, we categorized the adjacency of these neighbors with respect to the newly divided (daughter) cells. To this end, we inspected whether the neighbor of a neighbor-parent pair is adjacent to only one or both of the daughter cells.

To automate the procedure, we distinguished the divided daughter cells into the daughter closer to the center of the SAM, which we termed cell ‘A’ and the second daughter cell we named cell ‘B’. We labeled each neighbor cell in a neighbor-parent pair as class 0, 1, or 2, according to whether it is adjacent to only cell ‘A’ or only cell ‘B’, or to both. We then predicted the local topology excluding and including dividing neighbors using five feature sets, similar to the analysis above.

To distinguish neighbor-parent pairs that are adjacent to two dividing cells, we considered the difference of topological features between neighbor and dividing parental cells in addition to the parental topological properties as features (Fig. S1B). As a result, we obtained the following feature sets: unweighted topology, topological features from all weightings (topo), biological features (bio, consisting of surface area and perimeter from neighbor and parent, as well as the shared cell wall and distance between the two), the combination of all topological and biological features (topo+bio), and a reduced feature set (*r*<0.3) including only topological features with *r*<0.3 with all biological features. We performed training, validation and testing, and inspected the learning curves and estimated the information content of the used features, as specified in the analysis above for wild-type data using a linear kernel. Additionally, we tested the classifiers on the *ktn* SAM, and wild-type and ktn floral meristem data.

### Application of the classifiers for division event and local topology

To combine the predictions of division events and local topology changes, we used the previously developed classifiers and applied them to predict how the topology of the test plants would change. To this end, we selected the classifiers including both topological and biological features (based on validation performance), and applied them one after another on to the test tissues to generate the topology of the next time points. Here, the predictions were made for only one time step (24 h), as longer periods required us to estimate changes in the biological features as cells predicted to divide would not necessarily divide in the observed tissue one step later.

To arrive at the predicted cellular connectivity network, we determined the cells predicted to divide and the future adjacency of divided cells with their neighbors. Next, we repeated the following four steps for all cells predicted to divide at time t, starting with a random cell: (1) we removed the dividing cell along with the edges connecting the neighbors that is dividing; (2) we added the daughter cells representing the cell closer (cell A) and farther (cell B) away from the SAM center; and (3) we connected the daughter cells with their neighbors based on the prediction from the local topology classifier (Fig. 4A).

To evaluate the performance of the combined application of division and topology prediction, we calculated all unweighted topology features for the cells that are dividing in neither the predicted nor the observed topology. Next, we plotted the non-dividing cells observed against the predicted features, determined best linear fit and *r* for all unweighted topological properties. In addition, we divided all predicted non-dividing cells, randomly assigned labels to the neighbors of dividing cells depending on how they will be connected to the divided cells based on the training-validation set representation. We then repeated the correlation analysis from above 100 times (differently reconnecting topologies) and compared the correlations between predicted and random topology propagation using a one sample *t*-test (scipy.stats.ttest_1samp) and Benjamini-Hochberg testing correction.

To evaluate the local recreation of the topology around dividing cells, we also compared the first neighborhoods of cells dividing in the predicted and observed tissue of the test plant by calculating the percentage of correctly labeled neighbors. The distributions of predicted accuracies are compared with an estimated random labeling of the neighbors using Kolmogorov–Smirnov-Test (scipy.stats.ks_2samp).

## Supplementary Material

Supplementary information

Reviewer comments
