# Supplementary material for: Topological properties accurately predict cell division events and organization of shoot apical meristem in Arabidopsis thaliana
Source: Development. 2022 Aug 16;149(16):dev201024. doi: 10.1242/dev.201024 (PMC9481972; doi:10.1242/dev.201024)
Supplement: Supplementary information [file develop-149-201024-s1.pdf]

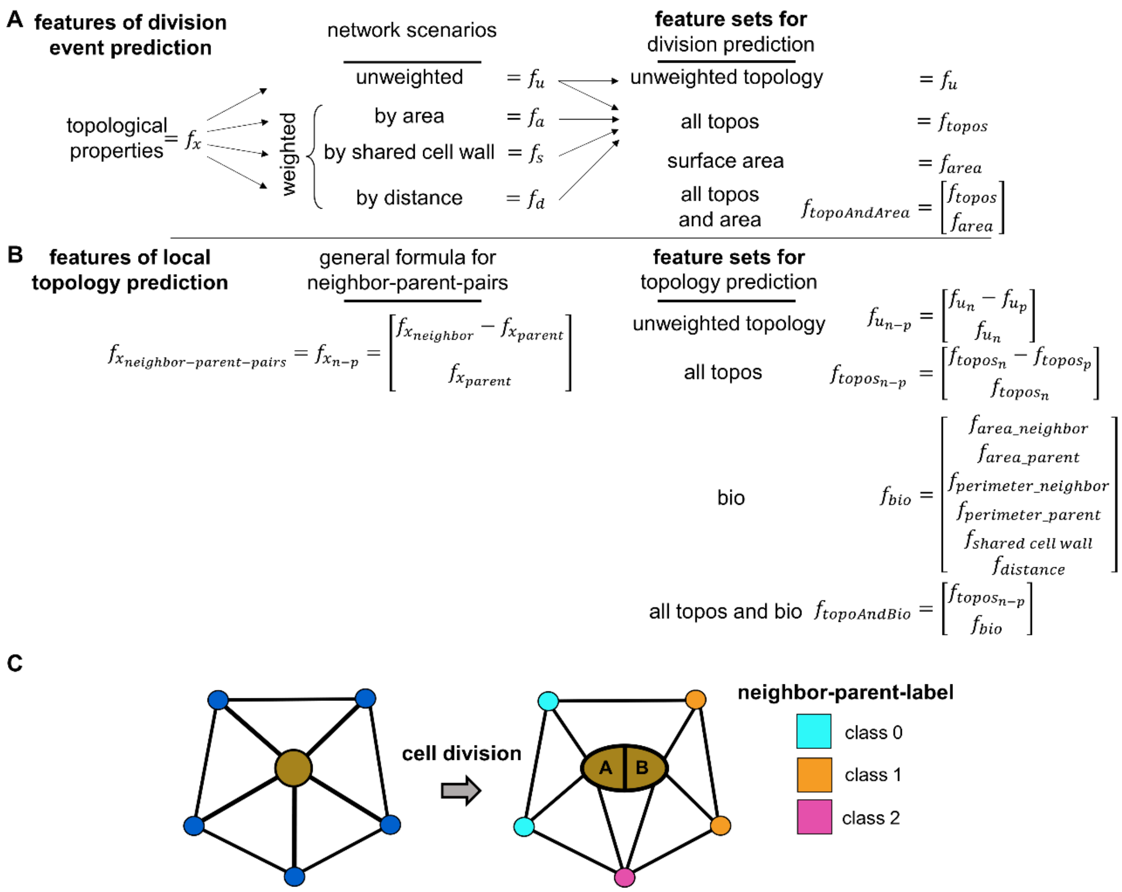

Fig. S1. Overview about feature sets for division event and local topology prediction as well as an example for local topology class assignment. (A) For division event prediction, 17 topological features (Supplementary Table 1) are calculated from four network scenarios (unweighted, weighted by area, shared cell wall, and distance, see Figure 1C), creating four different feature sets: unweighted topology (f_u), all topologies (f_{topos}), surface area (f_{area}), as well as topological features with surface area combined ($f_{toposAndArea}$) for all central cells. (B) For local topology prediction, the features are calculated for each neighbor-parent-pair of dividing parent cells using the difference in topological features of the neighbor ($f_{Xneighbor}$) and parent features ($f_{Xparent}$) as well as the features of the parent. Using this general formula, four feature sets are generated: unweighted topology, all topos, biological features (bio, including surface area, perimeter, shared cell wall, and distance), as well as topological and biological features combined (all topos and bio). (C) Parent cell (brown circle) divides into two daughter cells (A, B: representing the cell closer and farther away from the SAM center) changing the local topology in the process. The colors of the neighbors after division of the central cell represents the adjacency of the neighbor with the daughter cells: class 0 (cyan) neighbor is adjacent to cell A, class 1 (orange) pair neighbor is adjacent cell B, and class 2 (magenta) neighbors are adjacent to both cells. The classes are then used to predict local topological changes from the earlier time point.

Development • Supplementary information

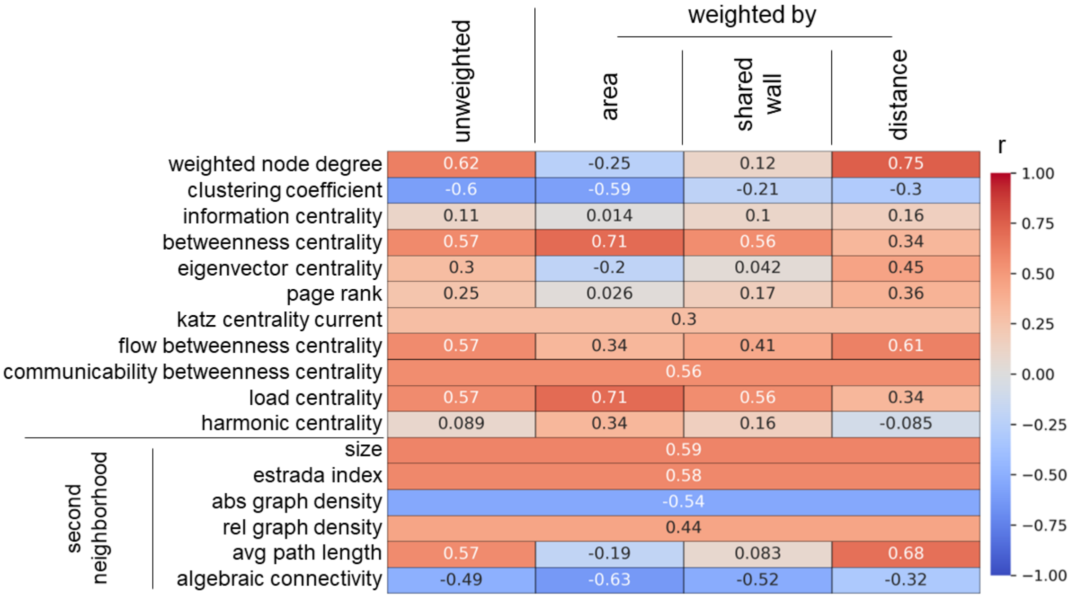

Fig. S2. Heat map of Pearson correlation coefficients between topological features and surface area. 17 topological features are calculated based on each of the four network scenarios (see Figure 1C): unweighted edges and edges weighted by area, shared wall, and distance. Majority of topological features exhibit small Pearson correlation coefficients (r, legend range from -1 (blue) to 1 (red)). N_{WT} = 20 tissue time steps, n_{WT} = 1445 cells.

Development • Supplementary information

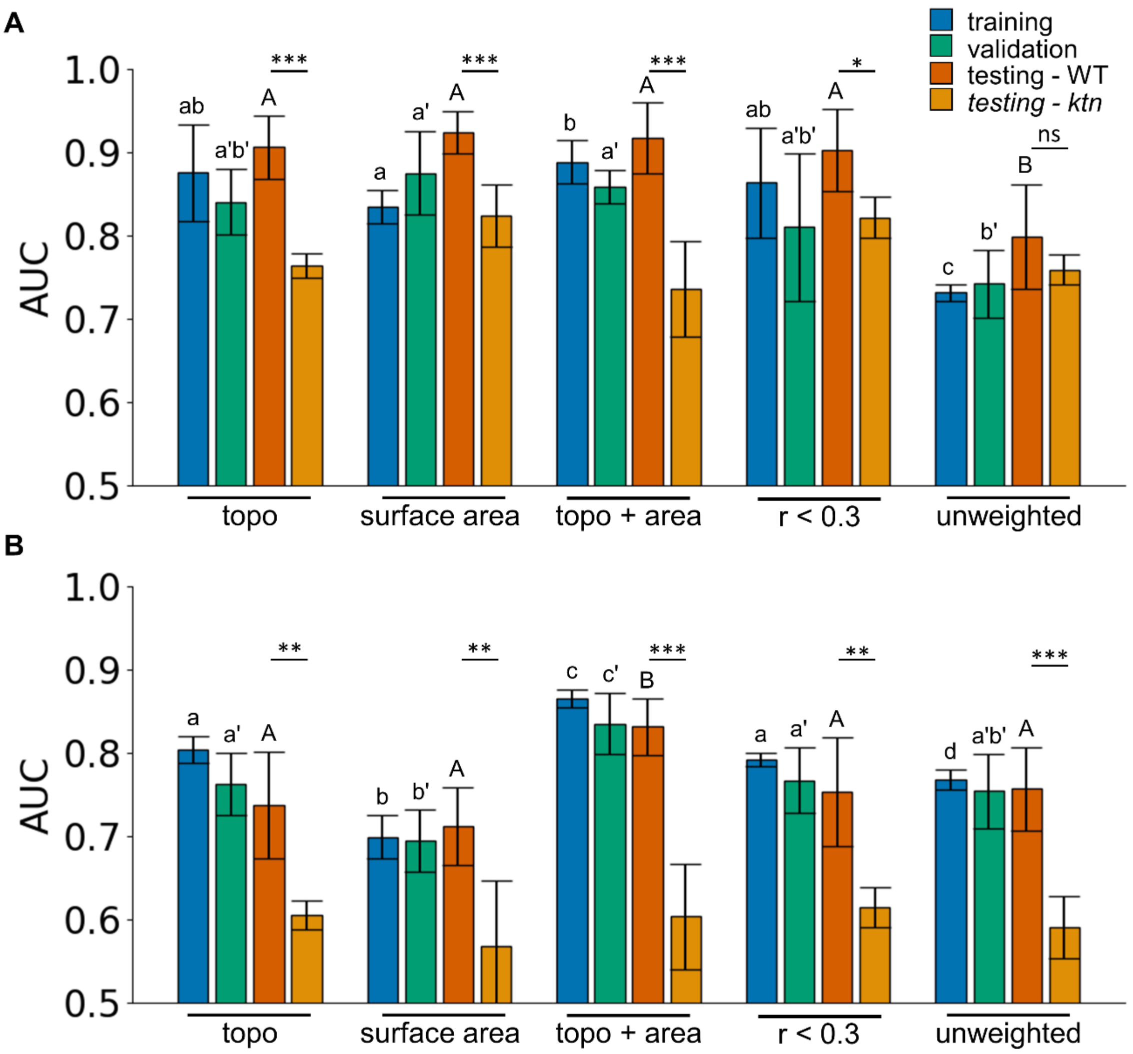

Fig. S3. Comparative analysis of predictions based using Area under the curve (AUC) of the ROC for feature sets. AUC of the ROC of the support vector machine (SVM) classifier on the training (blue), validation (green), and testing of wild type (orange) and *ktn* mutant (light orange) set of (A) division event and (B) local topology prediction. SVMs are trained on the combined topological features (topo), (A) surface area or (B) biological features (bio, including surface area, perimeter, shared cell wall, and distance), topological features with (A) surface area (topo + area) or (B) bio (topo + bio), reduced set of topological features that show an absolute Pearson correlation coefficient with (A) surface area or (B) bio smaller than 0.3 (r < 0.3), as well as only the topological features derived from the unweighted network scenario (unweighted). The performance on the training and validation set is determined from six-fold cross-validation with mean and the standard deviation shown as error bars. Different letters indicate significance between groups using Benjamini-Hochberg corrected two-tailed paired t-test (p-value < 0.05).

Statistical testing for differences of classifier performance for the training, validation and test sets was conducted separately (small letter without and with apostrophe and capital letter, respectively). Lines indicate Benjamini-Hochberg corrected two-tailed Student's t-test between test WT and *ktn* SAM with non-significant (ns), p-value < 0.05 (*), p-value < 0.01 (*), p-value < 0.001 (***) pairs. N_{WT} = 28 (20 tissue time steps for training-validation and 8 for testing); N_{ktn} = 5; (A) n_{WT} = 1445 and 618, train-validation and test cells respectively; n_{ktn} = 575 and (B) n_{WT} = 2103 and 912, train-validation and test cells, respectively; n_{ktn} = 1042. bars represent mean±s.d**.**

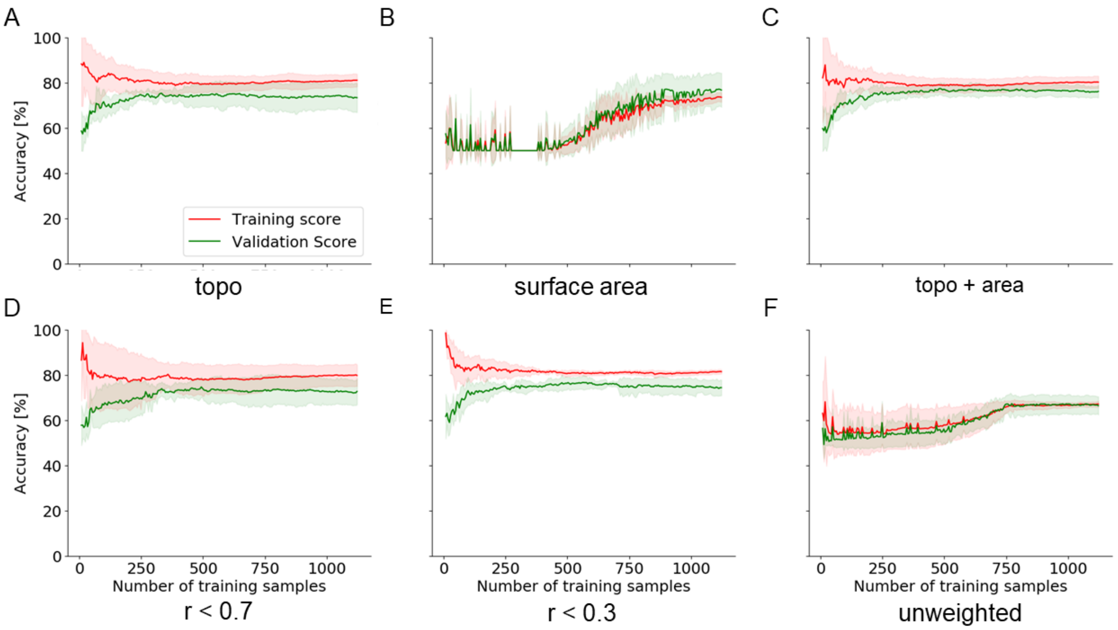

Fig. S4. Learning curves for the classifiers that predict division events. Learning curves of SVMs predicting cell division events, based on four different feature sets, showing the accuracy in the validation (green) and training (red) set (line: mean, area: ± 1 standard deviation). Feature sets: (A) combined topological features (topo, including features calculated from the four network scenarios, see Figure 1C), (B) surface area as a single feature, (C) topo with surface area (topo + area), (D, E) topological features, which have an absolute Pearson correlation coefficient (r) with surface area smaller than 0.3, respectively, and (F) unweighted topological features (unweighted topology). N_{WT} = 20 tissue time steps; n_{WT} = 1445 train-validation cells.

Development • Supplementary information

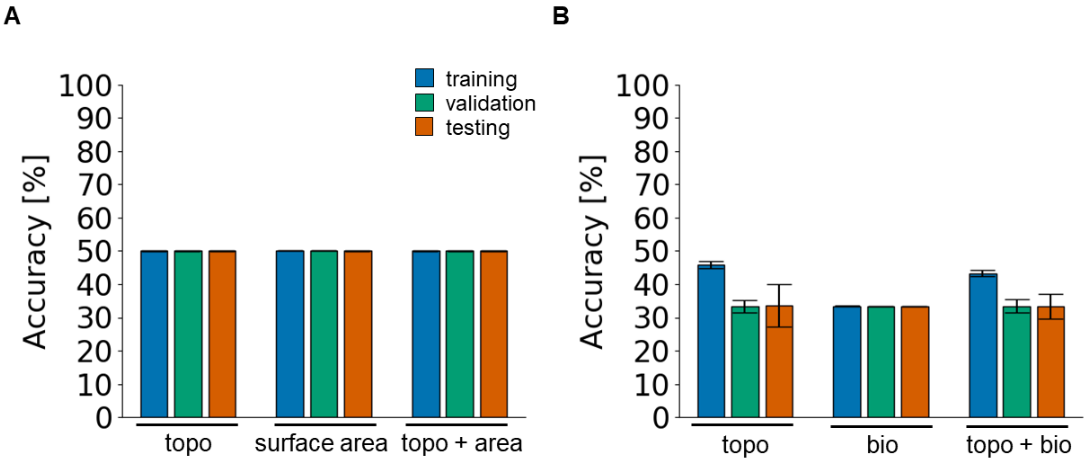

Fig. S5. Performance of classifiers trained on randomized labels. Support vector machines (SVMs) trained on surface area/biological features and unweighted topological features perform similarly with respect to the prediction of a division event and local topology of shoot apical meristems (SAM). Accuracy of the SVM classifier on the training (blue), validation (green), and testing (orange) set for a division event (A) and local topology prediction (B) based on unweighted topological features (unweighted topology), surface area, or biological features (bio). Shown are the mean and standard deviation on the training and validation sets from six-fold cross-validation. The performance on the training and validation set is determined from six-fold cross-validation with mean and the standard deviation shown as error bars. Different letters indicate significance between groups using paired t-test: p-value < 0.05. Statistical testing for differences of classifier performance for the training and validation sets was conducted separately (letter without and with apostrophe, respectively). N_{WT} = 28 (20 tissue time steps for training-validation and 8 for testing); N_{ktn} = 5; (A) n_{WT} = 1445 and 618, train-validation and test cells respectively; n_{ktn} = 575 and (B) n_{WT} = 2103 and 912, train-validation and test cells, respectively; n_{ktn} = 1042. bars represent mean±s.d**.**

Development • Supplementary information

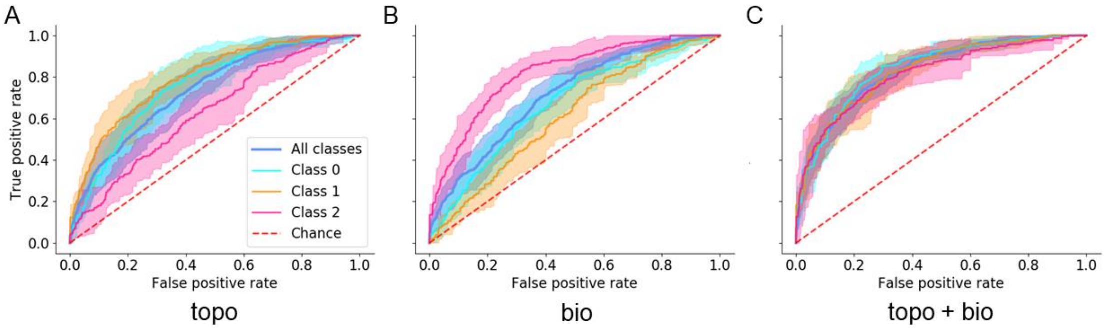

Fig. S6. Difference between classifiers trained on topological and biological features to predict local topology. Classifiers based on topological and biological features predict the three different classes of cells that are neighbors of cells that have divided in comparison to the previous time point. Receiver operating characteristic (ROC)-curve predicting cell division events on six-fold cross-validation of (A) combined topological features (topo, including features calculated from the four network scenarios, see Figure 1C), (B) biological features (bio, including surface area, perimeter, shared cell wall, and distance), and (C) topological and biological features combined (topo + bio). The mean performance is shown as a straight line, together with the area of ± 1 standard deviation obtained from the five-fold cross validation of the average ROC-curve combining all classes (blue), of class 0 (cyan), class 1 (orange), and class 2 (magenta). The performance expected by chance is marked with a red dashed line. N_{WT} = 8 test tissue time steps; n_{WT} = 912 test cells.

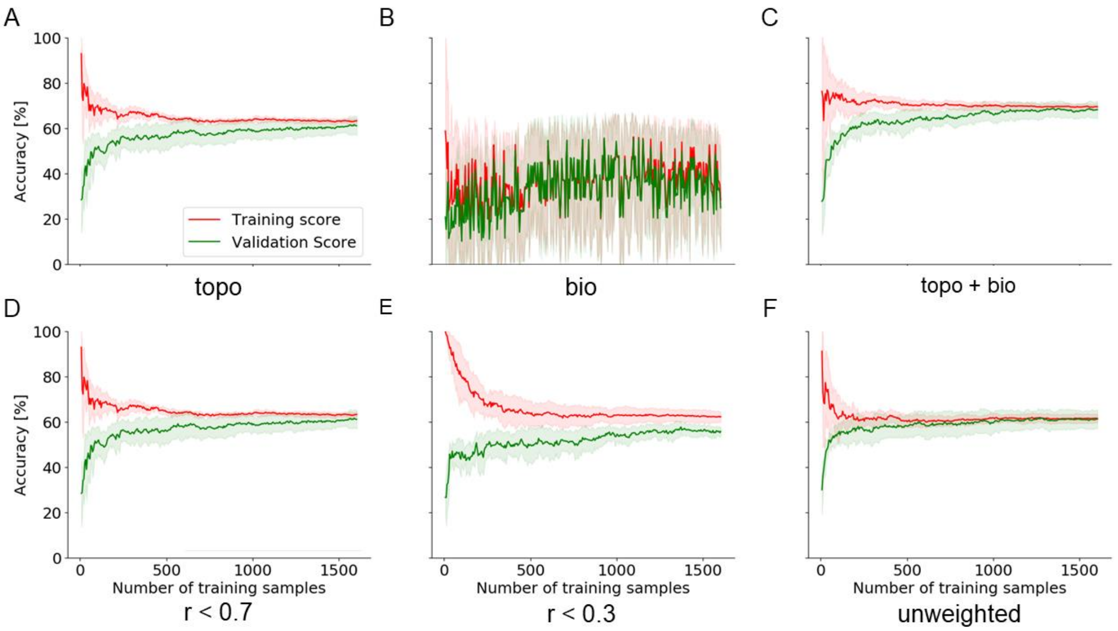

Fig. S7. Learning curves for the classifiers predicting local topological changes. Learning curves of support vector machines (SVMs) that predict local topology based on five different feature sets: (A) combined topological features (topo, including features calculated from the four network scenarios, see Figure 1C), (B) biological features (bio, including surface area, perimeter, shared cell wall, and distance), (C) topological and biological features combined (topo+bio), (D, E) topological features, which have an absolute value of Pearson correlation coefficients with all biological features (cor) smaller than 0.3, and (F) only unweighted features showing validation (green) and training (red) accuracy (line: mean, area: ± 1 standard deviation). N_{WT} = 20 tissue time steps; n_{WT} = 2103 train-validation cells.

Development • Supplementary information

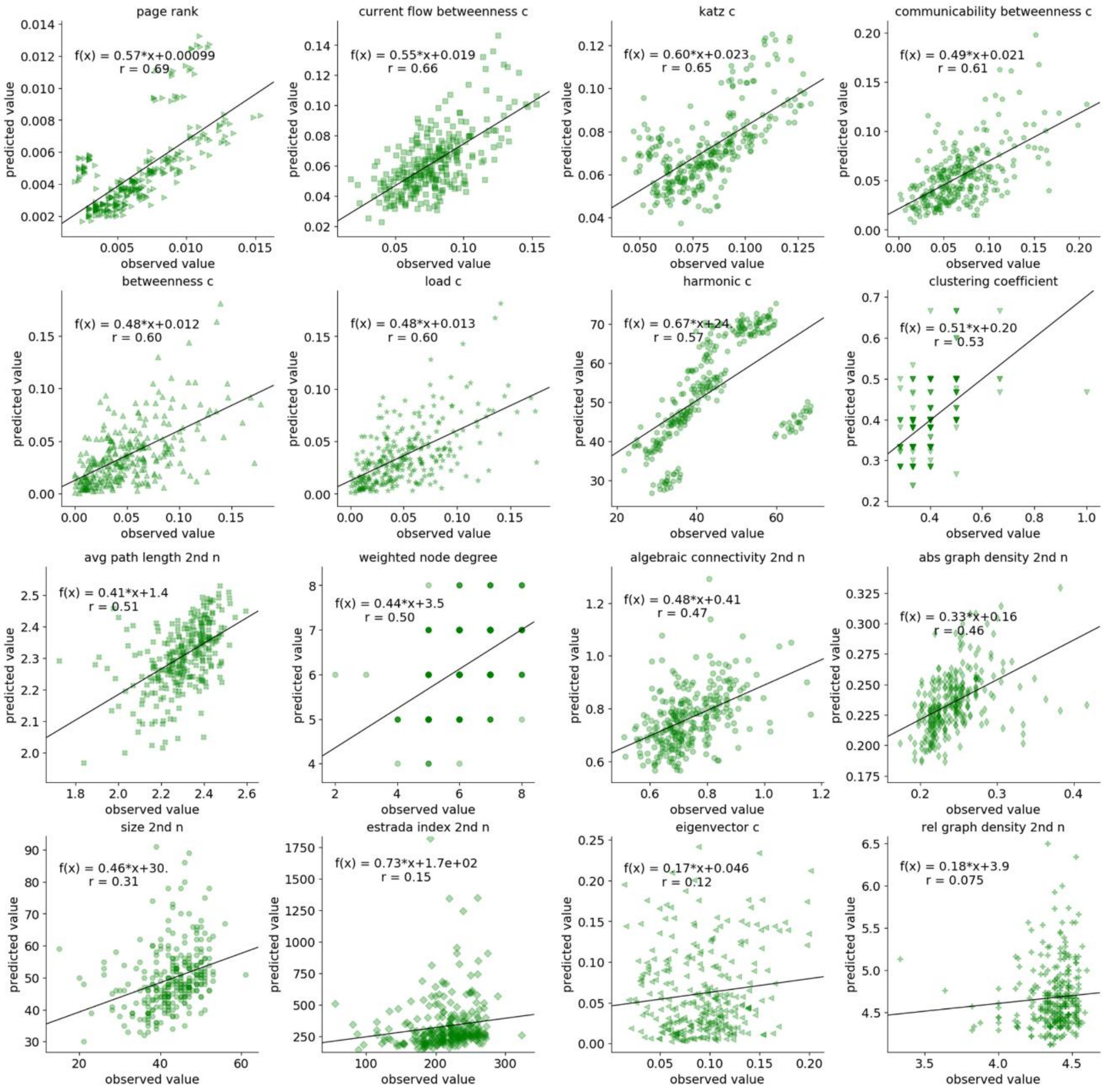

Fig. S8. Observed and predicted features from non-dividing cells plotted against each other. The observed feature values are plotted against the predicted features values for each non-dividing cell in the observed and predicted tissue. A line fitting the linear regression line is plotted to the data including its function f(x) and Pearson correlation coefficient r for the unweighted topological features: page rank, current flow betweenness c (centrality), katz c, communicability betweenness c, betweenness c, load c, harmonic c, clustering coefficient, average (avg) path length on 2 neighborhood (2nd n) , weighted node degree, algebraic connectivity 2nd n, absolute (abs) graph density 2nd n, size 2nd n, estrada index 2nd n, eigenvector c, relative (rel) graph density 2nd n. The predicted tissue is estimated using the division and topology prediction classifiers trained on topological and topological with biological features of the test tissues, respectively (see Figure 4E). N_{WT} = 8 tissue time steps, n_{WT} = 290 cells.

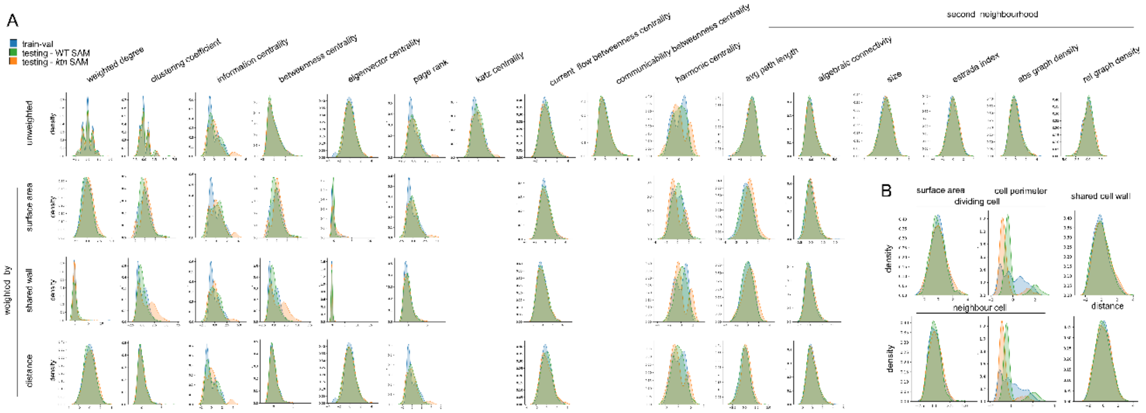

Fig. S9. Density distributions of topological and biological features of WT and ktn data. The density distributions of the normalized (A) topological and (B) biological features are plotted to differentiate between pooled samples used during training and validation from wild-type (WT; blue), and testing from WT (green), and ktn (orange). The topological features are grouped into unweighted and weighted by area, shared cell wall, and distance (see Figure 1C), while topological features independent of the network scenario are only displayed in the unweighted row. The columns are further grouped into features calculated on the second neighborhood and features from the dividing cell or its neighbor. (A) n_{WT} = 1445 and 618, train-validation and test cells respectively; n_{ktn} = 575 (B) n_{WT} = 2103 and 912, train-validation and test cells respectively; n_{ktn} = 1042.

Development • Supplementary information

Table S1. Definition of network centralities applied on differently weighted network scenarios to be used as feature sets for cell division and local topology prediction. Each network centrality is applied on the unweighted, weighted by area, shared cell wall, and distance network scenario. The network centralities were concatenated together for each cell and used to train and predict different classifiers.

Network centrality	Definition	Reference
weighted node degree	$_{D}(v) = \frac{\deg(v)}{\lvert V\rvert - 1}$	Freeman, 1979
clustering coefficient	$_{u} = \frac{1}{deg(u)(deg(u)-1))}\sum_{vw} \hat{w}_{uv}\hat{w}_{uw}\hat{w}_{vw}$	Saramäki, 2007
information centrality	$C_I(v) = \frac{\lvert V\rvert - 1}{\sum_{u \in V} p_{uv}(v) - p_{uv}(u)}$	Sabidussi, 1966
betweenness centrality	$C_B(v) = \frac{2}{(\lvert V\rvert - 1)(\lvert V\rvert - 2)}\sum_{s,t \in V}\frac{\sigma_v(s,t)}{\sigma(s,t)}$	Freeman, 1977
eigenvector centrality	$C_E(v) = \frac{1}{\lambda}\sum_{u \in V} a_{v,u}C_E(u)$	Bonacich, 1987
PageRank	$R'(u) = c\sum_{v \in B_u}\frac{R'(v)}{N_v} + cE(u)$	Page, 1999
Katz centrality	$x_i = \alpha\sum_j A_{ij} + \beta$	Katz, 1953
current flow betweenness centrality	$c_{CB}(v) = \frac{1}{(n-1)(n-2)}\sum_{s,t \in V}\tau_{st}(v)$	Newman, 2005
communicability betweenness centrality	$C_{CFB}(v) = \frac{2}{(\lvert V\rvert - 1)(\lvert V\rvert - 2)}\sum_{s,t \in V}\tau_{st}(v)$	Newman, 2005
load centrality	$_{L} = \frac{1}{(\lvert V\rvert - 1)(\lvert V\rvert - 2)}\sum_{s,t \in V}\theta_{s,t}(v)$	Goh, 2001
harmonic centrality	$C_H(v) = \frac{\sum_{u \in V}\frac{1}{d(u,v)}}{\lvert V\rvert - 1}$	Marchiori & Latora, 2000
size	number of edges in a graph	
Estrada index	$EEG(G) = \sum_{j=1}^{n} e^{\lambda_j}$	Estrada, 2005
absolute graph density	$= \frac{2m}{n(n-q)}$	Wilkis & Meara, 2002
relative graph density	$= \frac{2m}{n}$	
average path length	$a = \sum_{s,t \in V}\frac{d(s,t)}{n(n-1)}$	Mao & Zhang, 2013
algebraic connectivity	second smallest eigenvalue of Laplacian matrix	Fiedler, 1973

Development • Supplementary information

Table S2. Features ordered by Pearson correlation coefficient with area. Each network centrality was applied on the unweighted, weighted by area, shared cell wall, and distance network scenario. The Pearson correlation coefficient with area was calculated and were ordered from highest to lowest.

Feature name	Pearson correlation coefficient	p-value
weighted node degree weighted by distance	0.753377502	0
betweenness centrality weighted by area	0.707512442	1.0591074960896e-311
avg path length on 2 neighborhood weighted by distance	0.682352679	1.596195111956464e-281
weighted node degree	0.620070225	2.0669662274793384e-218
current flow betweenness centrality weighted by distance	0.611707287	4.812371330617275e-211
size on 2 neighborhood	0.594935365	5.057021194729162e-197
estrada index on 2 neighborhood	0.583528003	5.997153229184418e-188
betweenness centrality	0.5705616	6.373117152528312e-178
avg path length on 2 neighborhood	0.568504991	2.158701190828338e-176
current flow betweenness centrality	0.567399589	1.3876677872553513e-175
load centrality	0.565921643	1.6842614226438536e-174
communicability betweenness centrality	0.56005322	3.0531146350824394e-170
betweenness centrality weighted by shared wall	0.555983135	2.550625705762799e-167
eigenvector centrality weighted by distance	0.447873742	4.982402890283743e-102
rel graph density on 2 neighborhood	0.441796006	4.698909119359268e-99
current flow betweenness centrality weighted by shared wall	0.414482956	3.222728406558215e-86
page rank weighted by distance	0.359448329	1.0059855816964388e-63
harmonic centrality weighted by area	0.34300905	8.090933479758919e-58
current flow betweenness centrality weighted by area	0.341238842	3.2637221377844e-57
load centrality weighted by distance	0.339933021	9.025353613645715e-57
betweenness centrality weighted by distance	0.339062411	1.7603904997155677e-56
eigenvector centrality	0.300908478	2.868592129312282e-44
katz centrality	0.296715935	4.5579982830626784e-43
page rank	0.24762875	4.402906698088474e-30
page rank weighted by shared wall	0.170029847	9.015304252249096e-15
harmonic centrality weighted by shared wall	0.162714549	1.2198443119058207e-13
information centrality weighted by distance	0.162353154	1.3608683154738197e-13
weighted node degree weighted by shared wall	0.116561928	1.2480863722759725e-07
information centrality	0.113964977	2.343018162399504e-07
information centrality weighted by shared wall	0.102419688	3.4450652871124502e-06
harmonic centrality	0.089330332	5.2274644865479356e-05

Development • Supplementary information

weighted node degree weighted by distance	0.057524217	0.033774282
avg path length on 2 neighborhood weighted by shared wall	0.056763189	0.03620415
page rank weighted by area	0.037413208	0.167598817
information centrality weighted by area	0.035267233	0.19334169
avg path length on 2 neighborhood weighted by distance	0.0323718	0.232514552
eigenvector centrality weighted by shared wall	0.03031164	0.263614219
eigenvector centrality weighted by distance	-0.051181347	0.05897689
eigenvector centrality weighted by area	-0.203077703	3.82713434466663024e-14
avg path length on 2 neighborhood weighted by area	-0.214150561	1.361632834653018e-15
clustering coefficient weighted by shared wall	-0.236129215	1.0319617716515097e-18
weighted node degree weighted by area	-0.289769264	9.320458571568519e-28
algebraic connectivity on 2 neighborhood	-0.493010144	2.5317849899442773e-84
algebraic connectivity on 2 neighborhood weighted by shared wall	-0.535130612	9.326326866241923e-102
abs graph density on 2 neighborhood	-0.54315277	2.3001423114310698e-105
clustering coefficient	-0.580538544	1.5441792454417 56e-123
clustering coefficient weighted by distance	-0.584348465	1.5932034339322458e-125
algebraic connectivity on 2 neighborhood weighted by distance	-0.594101302	9.896429835985306e-131
algebraic connectivity on 2 neighborhood weighted by area	-0.639772476	1.1317137430247356e-157
clustering coefficient weighted by area	-0.642874421	1.1369324719240177e-159

Development • Supplementary information

Table S3. Performance measures of division event prediction from SVMs trained on features from four differently weighted topologies and/or area. F1-score (F1), accuracy (Acc), true positive rate (TPR), false positive rate (FPR), and area under the ROC-curve (Auc) of training (train) and unseen data (val) on the splits of the six-fold cross-validations, as well as retraining on train+val data testing on test data (testing), their mean and standard deviation (std) as well as the performance of testing on a never seen plant training on the full training-validation data set. Feature sets: combined topological features (topo; including features of unweighted, weighted by area-, shared cell wall-, and distance topologies), area as a single feature, topo with area, topological features which have an absolute Pearson correlation coefficient with area smaller than 0.3 (cor < 0.3) and unweighted topological features (unweighted).

topo

	train F1	train Acc	train precision	train Auc	val F1	val Acc	val precision	val Auc
split 0	85.07	84.63	82.68	0.91	43.38	61.01	79.19	0.81
split 1	83.36	82.90	81.16	0.91	80.85	76.32	67.86	0.90
split 2	83.91	83.62	82.42	0.91	70.79	73.56	79.06	0.83
split 3	75.64	75.85	76.29	0.82	79.12	76.25	70.59	0.84
split 4	81.57	80.89	78.76	0.89	79.90	80.08	80.65	0.86
split 5	82.21	81.79	80.35	0.89	79.20	77.43	73.45	0.86
train mean	81.96±3.04	81.61±2.84	80.28±2.21	0.89±0.03	72.21±13.32	74.11±6.17	75.13±4.81	0.85±0.03
test WT SAM	81.48±11.86	81.85±8.61	80.91±8.64	0.91±0.06	70.42±16.86	75.96±9.39	85.96±9.2	0.92±0.05
test ktn SAM					64.19±4.87	65.78±3.51	69.88±8.39	0.75±0.03
test WT floral					70.33±20.66	75.94±11.89	85.59±12.5	0.89±0.08
test ktn floral					65.16±12.61	72.77±6.44	91.65±13.37	0.9±0.05

area

	train F1	train Acc	train precision	train Auc	val F1	val Acc	val precision	val Auc
split 0	64.72	69.84	77.94	0.82	88.00	87.78	86.45	0.93
split 1	73.20	75.66	81.42	0.85	78.20	76.34	72.51	0.83
split 2	74.83	76.98	82.53	0.87	50.26	63.76	80.11	0.79
split 3	72.77	74.00	76.37	0.82	81.01	81.25	82.05	0.89
split 4	72.35	74.39	78.61	0.82	78.95	80.00	83.33	0.91
split 5	66.45	71.95	82.66	0.83	55.03	68.07	93.06	0.90
train mean	70.72±3.75	73.8±2.35	79.92±2.41	0.83±0.02	71.91±14.05	76.2±8.11	82.92±6.22	0.88±0.05
test WT SAM	76.74±14.89	79.83±9.36	84.16±8.02	0.91±0.05	72.54±16.77	78.3±8.64	90.45±7.93	0.92±0.02
test ktn SAM					66.78±8.76	71.43±4.70	78.43±3.45	0.82±0.04
test WT floral					67.21±14.66	75.24±8.54	93.49±7.19	0.93±0.04
test ktn floral					55.75±14.00	69.87±6.97	98.8±3.40	0.93±0.03

topo with area

Development • Supplementary information

	train F1	train Acc	train precision	train Auc	val F1	val Acc	val precision	val Auc
split 0	82.79	82.20	80.16	0.89	69.27	74.65	87.92	0.87
split 1	82.28	81.99	80.98	0.91	81.43	77.19	68.67	0.89
split 2	83.88	83.50	82.02	0.91	70.05	72.90	78.29	0.83
split 3	75.99	76.30	77.00	0.83	79.70	77.08	71.52	0.85
split 4	81.23	80.76	79.31	0.89	78.71	78.58	78.25	0.86
split 5	82.04	81.57	80.01	0.90	75.75	74.98	73.51	0.85
train mean	81.37±2.54	81.06±2.28	79.91±1.56	0.89±0.03	75.82±4.67	75.90±1.90	76.36±6.21	0.86±0.02
test WT SAM	81.71±11.89	81.98±8.61	80.64±8.49	0.91±0.06	71.78±16.57	76.75±8.94	85.76±8.95	0.92±0.04
test ktn SAM					64.86±5.92	66.75±2.47	70.26±7.28	0.74±0.03
test WT floral					72.16±18.17	77.33±10.81	87.32±11.27	0.89±0.08
test ktn floral					69.88±10.61	76.22±6.58	94.97±10.74	0.91±0.05

cor < 0.3

	train F1	train Acc	train precision	train Auc	val F1	val Acc	val precision	val Auc
split 0	83.74	83.21	81.19	0.89	56.72	67.30	83.83	0.83
split 1	81.72	81.29	79.88	0.90	80.85	76.32	67.86	0.91
split 2	83.02	82.22	79.48	0.90	70.64	72.49	75.73	0.82
split 3	82.62	82.08	80.22	0.88	78.74	77.50	74.63	0.87
split 4	81.26	80.73	79.12	0.89	75.17	74.54	73.35	0.83
split 5	81.81	81.22	79.33	0.89	79.40	78.11	74.98	0.87
train mean	82.36±0.85	81.79±0.82	79.87±0.69	0.89±0.01	73.59±8.25	74.38±3.68	75.06±4.70	0.85±0.03
test WT SAM	81.08±9.31	81.28±7.01	81.13±8.36	0.91±0.06	71.31±19.30	76.43±11.50	84.16±11.85	0.90±0.05
test ktn SAM					69.25±11.41	69.83±6.35	71.36±7.96	0.82±0.03
test WT floral					63.41±27.92	72.62±11.45	69.92±27.19	0.87±0.05
test ktn floral					59.27±15.65	69.73±6.99	91.13±14.29	0.87±0.07

unweighted

	train F1	train Acc	train precision	train Auc	val F1	val Acc	val precision	val Auc
split 0	68.40	69.52	71.00	0.76	59.70	61.42	62.49	0.68
split 1	65.15	66.61	68.14	0.74	65.14	64.33	63.69	0.69
split 2	67.92	68.84	70.00	0.75	57.19	62.58	66.80	0.71
split 3	63.39	66.20	69.14	0.73	76.61	73.54	68.65	0.78
split 4	64.66	65.56	66.38	0.72	72.46	71.50	70.09	0.81
split 5	65.76	66.29	66.82	0.73	63.47	64.93	66.23	0.72
train mean	65.88±1.77	67.17±1.47	68.58±1.65	0.74±0.01	65.76±6.81	66.38±4.52	66.33±2.63	0.73±0.05
test WT SAM	68.83±10.35	70.22±7.12	70.79±6.23	0.77±0.09	68.35±12.59	69.76±8.43	71.23±9.47	0.80±0.06
test ktn SAM					68.06±6.35	68.88±3.96	69.15±1.30	0.76±0.02
test WT floral					60.51±6.18	65.36±6.23	74.31±15.6	0.76±0.09
test ktn floral					58.23±13.71	66.38±8.72	75.45±14.33	0.74±0.10

Development • Supplementary information

Table S4. Performance measures of local topology prediction from SVMs trained from four different topologies and/or biological features. F1-score (F1), accuracy (Acc), true positive rate (TPR), false positive rate (FPR), and area under the ROC-curve (Auc) of training (train) and unseen data (val) on the splits of the six-fold cross-validations, as well as retraining on train+val data testing on test data (testing) (A) excluding or (B) including dividing neighbors, their mean and standard deviation (dev) as well as the performance of testing on a never seen plant training on the full training-validation data set. Feature sets: combined topological features (topo; including features of unweighted, weighted by area-, shared cell wall-, and distance topologies), biological features (bio; including area, perimeter, shared cell wall, and distance), topo with bio, topological features which have an absolute value of the Pearson correlation coefficients with all biological features smaller than 0.3 (cor < 0.3), respectively, and unweighted topological features (unweighted).

A

topo

	train F1	train c0 F1	train c1 F1	train c2 F1	train Acc	train precision	train c0 precision	train c1 precision	train c2 precision	train Auc	val F1	val c0 F1	val c1 F1	val c2 F1	val Acc	val precision	val c0 precision	val c1 precision	val c2 precision	val Auc
split 0	66.81	69.10	72.48	58.84	66.94	66.75	67.71	71.53	61.02	0.82	51.25	53.90	55.04	44.82	51.30	51.66	49.78	59.32	45.90	0.71
split 1	62.47	62.96	68.53	55.94	62.53	62.47	64.31	66.98	56.11	0.80	62.21	65.99	71.30	49.35	62.69	62.19	67.78	66.12	52.67	0.81
split 2	65.77	67.89	72.70	56.73	65.94	65.69	66.89	71.31	58.86	0.83	51.66	55.63	53.42	45.92	51.70	51.77	53.20	56.01	46.10	0.72
split 3	60.81	62.26	66.72	53.44	60.94	60.75	62.57	64.72	54.96	0.79	58.68	61.73	63.46	50.86	58.43	59.71	58.85	71.85	48.42	0.78
split 4	62.81	64.84	68.51	55.08	62.95	62.75	64.74	66.58	56.92	0.80	59.56	58.62	68.68	51.38	59.85	59.47	58.65	65.53	54.23	0.78
split 5	61.58	62.90	66.82	55.01	61.60	61.55	63.04	66.26	55.36	0.79	60.74	60.52	66.15	55.57	60.93	61.48	68.43	60.58	55.44	0.77
train mean	63.38±2.18	64.99±2.62	69.29±2.44	55.84±1.68	63.48±2.21	63.33±2.17	64.88±1.88	67.90±2.59	57.21±2.12	0.80±0.02	57.35±4.31	59.4±3.98	63.01±6.66	49.65±3.58	57.48±4.42	57.71±4.34	59.45±6.87	63.23±5.20	50.46±3.83	0.76±0.04
test WT SAM	63.00±7.77	64.83±8.97	68.64±11.74	55.53±8.14	63.53±7.69	64.16±7.07	65.37±8.55	69.25±7.87	57.86±9.99	0.81±0.06	51.51±8.97	53.65±18.46	60.21±8.55	40.67±9.82	53.29±7.64	53.73±7.32	54.59±6.47	61.47±8.77	45.12±9.79	0.74±0.06
test ktn SAM											43.54±3.50	44.16±4.50	41.22±8.61	45.24±4.16	44.01±3.21	44.54±3.38	42.99±4.84	43.73±4.68	46.92±6.27	0.61±0.02
test WT floral											45.94±7.59	48.92±5.20	39.64±17.2	49.27±3.49	46.91±6.26	46.96±7.0	47.66±8.39	45.48±14.94	47.73±3.60	0.66±0.07
test ktn floral											42.91±6.34	47.38±6.51	36.46±12.78	44.88±4.11	44.15±5.31	45.85±4.69	44.44±6.72	50.57±8.46	42.52±5.36	0.62±0.06

bio

	train F1	train c0 F1	train c1 F1	train c2 F1	train Acc	train precision	train c0 precision	train c1 precision	train c2 precision	train Auc	val F1	val c0 F1	val c1 F1	val c2 F1	val Acc	val precision	val c0 precision	val c1 precision	val c2 precision	val Auc
split 0	42.32	2.27	55.01	69.67	51.18	49.84	41.40	42.43	65.68	0.67	38.31	2.00	51.64	61.28	46.37	44.54	34.01	39.11	60.49	0.64
split 1	48.35	23.41	53.67	67.97	52.30	55.77	58.68	42.77	65.85	0.71	48.62	29.03	47.49	69.34	50.45	50.77	44.98	39.97	67.38	0.72
split 2	54.37	51.31	44.21	67.59	55.02	54.21	52.16	47.95	62.51	0.74	52.91	53.99	36.11	68.62	54.53	53.42	49.51	47.63	63.13	0.76
split 3	52.09	38.10	50.53	67.65	53.27	54.52	54.84	43.37	65.36	0.72	44.78	15.45	51.66	67.22	49.79	50.69	46.33	40.78	64.95	0.70
split 4	41.60	3.86	53.97	66.97	49.79	51.33	48.24	41.39	64.37	0.67	43.83	2.00	56.18	73.30	53.46	70.11	100.00	44.16	66.18	0.68
split 5	42.08	3.28	54.68	68.29	50.47	48.76	38.80	41.98	65.50	0.69	40.46	4.07	51.51	65.80	48.19	49.73	46.29	39.48	63.43	0.68
train mean	46.80±5.12	20.37±19.03	52.01±3.78	68.02±0.84	52.00±1.77	52.40±2.58	49.02±7.07	43.32±2.16	64.88±1.16	0.70±0.03	44.82±4.87	17.76±18.84	49.10±6.33	67.59±3.65	50.46±2.83	53.21±8.01	53.52±21.35	41.85±3.07	64.26±2.24	0.69±0.04
test WT SAM	52.92±4.88	46.80±7.81	43.06±8.37	68.90±7.58	53.93±4.89	53.46±5.19	50.30±7.93	45.17±6.24	64.91±7.03	0.74±0.05	48.23±4.05	38.72±10.84	40.23±9.50	65.76±6.54	50.16±4.88	49.60±5.43	45.54±10.80	42.65±7.24	60.62±3.58	0.71±0.05
test ktn SAM											40.02±7.25	34.72±9.57	39.26±4.76	46.08±12.19	40.45±7.25	40.28±7.16	37.52±7.80	40.82±6.40	42.51±12.20	0.57±0.08
test WT floral											52.35±4.51	44.69±5.19	39.41±11.24	72.95±4.19	53.75±4.59	53.12±5.80	47.80±8.75	42.41±6.90	69.15±6.74	0.75±0.03
test ktn floral											50.69±2.97	34.95±10.66	41.48±10.73	75.64±4.06	52.96±3.14	51.43±3.37	41.00±6.85	42.39±3.66	70.91±6.91	0.74±0.03

topoAndBio

	train F1	train c0 F1	train c1 F1	train c2 F1	train Acc	train precision	train c0 precision	train c1 precision	train c2 precision	train Auc	val F1	val c0 F1	val c1 F1	val c2 F1	val Acc	val precision	val c0 precision	val c1 precision	val c2 precision	val Auc
split 0	71.76	70.59	70.65	74.02	71.74	71.82	68.75	72.01	74.71	0.87	58.17	55.48	56.45	62.58	58.31	58.27	57.48	58.35	58.97	0.77
split 1	72.38	71.00	72.35	73.80	72.37	72.41	70.42	71.97	74.83	0.87	76.77	73.70	81.10	75.50	76.94	77.65	73.13	74.72	85.09	0.90
split 2	74.09	72.16	75.79	74.33	74.10	74.10	71.95	75.23	75.12	0.88	63.47	63.51	58.73	68.16	63.58	64.00	58.46	64.51	69.02	0.82
split 3	68.97	67.43	67.72	71.76	68.95	69.01	66.57	67.58	72.89	0.85	67.44	68.82	64.68	68.82	67.61	68.79	61.48	75.08	69.81	0.84
split 4	71.97	70.39	72.66	72.85	71.97	72.00	70.82	71.07	74.13	0.87	65.08	60.03	66.46	68.74	65.21	65.65	65.28	60.71	70.95	0.85
split 5	69.95	68.39	69.29	72.19	69.95	69.97	67.47	70.13	72.33	0.86	63.63	62.45	62.39	66.04	63.60	63.95	65.62	58.62	67.60	0.83
train mean	71.52±1.66	69.99±1.60	71.41±2.59	73.16±0.96	71.51±1.67	71.55±1.66	69.33±1.90	71.33±2.30	74.00±1.04	0.86±0.01	65.76±5.66	64.00±5.89	64.97±7.97	68.31±3.88	65.88±5.68	66.38±5.93	63.57±5.26	65.33±7.06	70.24±7.71	0.84±0.04
test WT SAM	71.89±9.01	70.12±11.09	70.84±13.78	74.70±8.27	72.12±8.71	72.40±9.04	70.17±10.77	70.69±11.55	76.34±9.87	0.87±0.05	64.26±5.48	64.39±10.76	64.92±6.61	63.49±7.32	64.79±5.17	65.64±5.24	64.62±4.33	67.14±10.58	65.16±6.00	0.83±0.03
test ktn SAM											41.24±5.25	42.86±4.19	44.08±7.07	36.79±7.05	41.66±5.41	42.66±6.78	40.31±5.24	45.08±3.84	42.59±13.55	0.60±0.06
test WT floral											59.58±8.83	55.18±10.86	53.14±14.40	70.42±3.59	60.08±8.29	60.45±9.08	56.35±12.53	56.36±13.54	68.63±7.79	0.79±0.07
test ktn floral											60.79±6.51	56.81±7.75	53.76±9.90	71.80±3.79	61.41±6.24	61.53±6.78	56.15±7.85	61.02±10.30	67.42±6.01	0.78±0.05

r < 0.3

Development • Supplementary information

	train F1	train c0 F1	train c1 F1	train c2 F1	train Acc	train precision	train c0 precision	train c1 precision	train c2 precision	train Auc	val F1	val c0 F1	val c1 F1	val c2 F1	val Acc	val precision	val c0 precision	val c1 precision	val c2 precision	val Auc
split 0	62.57	63.01	69.35	55.34	62.60	62.54	63.13	68.74	55.73	0.80	52.55	53.88	57.45	46.31	52.44	52.71	54.16	58.97	44.99	0.71
split 1	61.95	64.28	68.02	53.54	62.09	61.88	64.42	66.00	55.21	0.79	64.49	68.86	73.98	50.63	65.07	64.34	66.48	70.88	55.66	0.83
split 2	62.12	63.88	69.57	52.93	62.30	62.04	64.46	67.18	54.49	0.81	53.90	55.54	56.43	49.72	53.89	53.93	54.45	57.58	49.77	0.72
split 3	59.56	61.12	66.65	50.92	59.83	59.49	60.61	63.90	53.97	0.78	61.00	65.92	66.75	50.34	61.11	61.67	60.19	73.18	51.63	0.79
split 4	60.85	62.25	67.77	52.55	61.03	60.78	62.43	65.50	54.40	0.78	60.25	60.68	65.82	54.26	60.36	60.22	59.78	64.60	56.28	0.78
split 5	61.18	63.04	67.42	53.09	61.28	61.12	63.03	66.07	54.25	0.79	59.74	57.04	68.46	53.74	59.99	60.95	68.35	63.63	50.86	0.77
train mean	61.37±0.99	62.93±1.04	68.13±1.03	53.06±1.31	61.52±0.94	61.31±1.00	63.01±1.31	66.23±1.49	54.68±0.60	0.79±0.01	58.66±4.15	60.32±5.47	64.81±6.15	50.83±2.65	58.81±4.34	58.97±4.21	60.57±5.40	64.81±5.70	51.53±3.79	0.77±0.04
test WT SAM	60.58±9.62	61.23±12.04	66.67±12.77	53.83±10.76	61.18±9.50	61.52±9.22	62.36±10.97	65.29±10.33	56.91±11.63	0.78±0.08	53.82±8.58	54.50±18.26	63.16±8.82	43.79±10.23	56.02±7.60	57.63±9.45	54.10±5.67	61.20±10.86	57.60±15.82	0.75±0.07
test ktn SAM											44.46±2.99	45.22±6.94	42.17±7.75	45.99±3.86	44.89±3.17	45.06±2.96	45.06±5.32	43.10±3.84	47.03±5.74	0.62±0.02
test WT floral											48.11±8.21	46.22±10.10	47.72±11.51	50.39±7.13	48.39±8.17	49.03±8.15	49.12±11.75	51.21±10.37	46.76±5.87	0.67±0.09
test ktn floral											47.17±6.91	49.01±10.33	44.24±10.71	48.25±3.87	47.79±6.46	48.45±7.27	48.91±9.10	51.02±10.94	45.41±4.75	0.65±0.07

unweighted

	train F1	train c0 F1	train c1 F1	train c2 F1	train Acc	train precision	train c0 precision	train c1 precision	train c2 precision	train Auc	val F1	val c0 F1	val c1 F1	val c2 F1	val Acc	val precision	val c0 precision	val c1 precision	val c2 precision	val Auc
split 0	60.84	64.14	65.99	52.38	60.91	60.79	63.08	65.94	53.35	0.78	48.03	45.25	57.32	41.53	48.69	48.58	51.73	50.65	43.35	0.68
split 1	58.04	59.69	64.49	49.93	58.21	58.02	61.11	61.53	51.42	0.76	60.07	67.31	68.67	44.22	60.96	59.92	64.81	64.38	50.56	0.81
split 2	60.48	65.46	67.10	48.87	60.99	60.43	63.59	63.69	54.01	0.79	51.23	55.04	52.44	46.22	51.27	51.39	52.57	55.57	46.04	0.71
split 3	57.54	58.98	62.56	51.09	57.53	57.56	59.91	62.02	50.75	0.75	56.77	63.29	66.98	40.05	57.86	56.63	59.53	63.23	47.11	0.79
split 4	58.49	61.48	64.42	49.55	58.70	58.40	61.05	62.22	51.93	0.77	60.58	64.56	66.95	50.23	60.99	60.55	63.50	63.43	54.72	0.77
split 5	58.88	61.72	64.52	50.41	59.07	58.82	61.82	62.05	52.60	0.77	59.34	64.37	65.32	48.32	59.80	59.30	62.85	61.91	53.14	0.77
train mean	59.04±1.22	61.91±2.28	64.85±1.42	50.37±1.13	59.24±1.30	59.00±1.20	61.76±1.26	62.91±1.51	52.34±1.11	0.77±0.01	56.00±4.75	59.97±7.60	62.95±5.96	45.10±3.58	56.59±4.85	56.06±4.54	59.17±5.21	59.86±5.03	49.15±4.01	0.75±0.04
test WT SAM	56.38±8.67	59.30±12.24	61.59±15.10	48.26±10.08	57.59±8.43	57.56±8.60	60.01±9.22	60.29±12.26	52.38±9.73	0.76±0.08	55.25±6.87	59.46±12.04	63.50±7.20	42.81±9.51	56.57±7.43	57.42±7.85	59.37±8.32	61.12±3.83	51.76±15.42	0.76±0.05
test ktn SAM											40.39±5.26	43.52±4.97	44.02±5.17	33.65±11.32	41.51±4.82	41.77±4.67	40.67±4.49	40.13±4.55	44.52±5.92	0.59±0.04
test WT floral											46.60±8.12	45.10±9.37	47.98±11.46	46.73±8.00	46.75±8.06	47.44±8.11	47.03±10.66	50.04±12.66	45.24±6.12	0.66±0.07
test ktn floral											49.88±6.83	49.71±10.13	51.83±10.18	48.10±3.81	50.04±6.74	50.32±6.84	50.96±8.43	54.41±10.29	45.59±3.71	0.67±0.07

B

topo

	train F1	train c0 F1	train c1 F1	train c2 F1	train Acc	train precision	train c0 precision	train c1 precision	train c2 precision	train Auc	val F1	val c0 F1	val c1 F1	val c2 F1	val Acc	val precision	val c0 precision	val c1 precision	val c2 precision	val Auc
split 0	61.68	64.86	67.47	52.70	62.02	61.71	62.29	65.28	57.54	0.79	51.17	55.35	59.84	38.32	52.30	51.71	51.90	54.79	48.44	0.73
split 1	60.28	63.41	66.50	50.94	60.67	60.30	61.42	63.57	55.92	0.78	61.87	68.37	65.97	51.27	61.92	61.89	69.82	63.85	52.01	0.80
split 2	64.63	68.64	70.61	54.62	64.95	64.58	66.09	68.84	58.82	0.81	54.35	58.22	56.28	48.57	54.38	54.49	55.75	59.20	48.53	0.73
split 3	59.59	63.44	65.08	50.24	59.92	59.56	61.06	63.17	54.44	0.78	59.49	64.72	68.42	45.33	60.37	59.81	58.66	67.28	53.50	0.80
split 4	59.83	64.03	65.89	49.58	60.27	59.82	61.63	63.22	54.61	0.78	61.56	65.44	69.89	49.34	62.66	62.61	61.89	63.46	62.47	0.80
split 5	60.10	64.23	65.93	50.12	60.54	60.13	62.47	62.46	55.48	0.78	61.60	62.66	65.81	56.34	61.55	61.81	65.86	64.97	54.58	0.78
train mean	61.02±1.75	64.77±1.80	66.91±1.80	51.37±1.76	61.40±1.72	61.02±1.73	62.49±1.68	64.42±2.15	56.13±1.57	0.79±0.01	58.34±4.12	62.46±4.43	64.37±4.79	48.20±5.52	58.86±4.01	58.72±4.14	60.65±6.02	62.26±4.12	53.26±4.72	0.77±0.03
test WT SAM	60.22±5.09	65.18±5.73	66.67±6.58	48.80±8.26	61.04±5.09	60.93±5.17	62.78±5.86	63.60±6.22	56.40±8.04	0.79±0.04	56.24±7.47	62.04±6.83	60.30±9.89	46.37±10.79	56.92±6.89	56.68±7.61	59.20±4.68	61.61±9.65	49.22±10.42	0.77±0.03
test ktn SAM											43.50±3.27	46.34±2.64	46.25±4.47	37.91±5.57	44.11±3.17	44.74±3.29	43.90±5.65	44.15±3.34	46.17±3.67	0.62±0.02
test WT floral											47.47±7.39	50.76±7.31	44.87±16.48	46.80±3.20	48.30±6.49	48.14±7.38	48.26±7.61	49.03±13.77	47.14±2.29	0.66±0.08
test ktn floral											47.33±4.58	50.80±6.94	45.10±6.57	46.10±3.01	47.67±4.48	48.29±4.61	48.33±6.52	52.02±7.08	44.52±2.40	0.67±0.05

bio

	train F1	train c0 F1	train c1 F1	train c2 F1	train Acc	train precision	train c0 precision	train c1 precision	train c2 precision	train Auc	val F1	val c0 F1	val c1 F1	val c2 F1	val Acc	val precision	val c0 precision	val c1 precision	val c2 precision	val Auc
split 0	48.28	56.31	18.45	70.07	53.34	53.81	44.91	49.31	67.21	0.75	45.43	56.47	11.80	68.02	51.87	52.61	43.70	47.13	67.01	0.73
split 1	42.27	2.64	55.04	69.12	50.88	46.20	30.57	42.48	65.57	0.70	42.86	3.70	56.55	68.31	51.55	70.05	100.00	42.74	67.42	0.70
split 2	53.02	47.57	42.49	69.00	53.62	52.72	48.49	45.02	64.66	0.74	52.68	45.81	41.56	70.68	53.70	52.41	46.45	46.33	64.46	0.75
split 3	42.33	1.47	55.74	69.77	51.41	48.09	34.95	42.70	66.63	0.69	40.33	0.00	55.09	65.90	49.54	35.38	0.00	41.68	64.45	0.67
split 4	43.25	6.07	54.86	68.83	51.01	49.63	41.00	42.64	65.24	0.70	42.73	0.00	56.07	72.12	52.36	37.00	0.00	43.12	67.88	0.72
split 5	42.67	3.24	55.59	69.19	51.34	49.42	40.12	43.04	65.10	0.71	41.92	3.57	54.46	67.72	50.51	59.44	72.76	42.11	63.45	0.70
train mean	45.30±4.03	19.55±23.08	47.03±13.61	69.33±0.44	51.93±1.11	49.98±2.60	40.01±5.94	44.20±2.44	65.73±0.89	0.72±0.02	44.32±4.03	18.26±23.50	45.92±16.11	68.79±2.04	51.59±1.32	51.15±12.10	43.82±36.15	43.85±2.10	65.78±1.71	0.71±0.02
test WT SAM	42.05±2.55	1.52±3.50	55.59±2.81	69.04±4.94	51.10±2.79	42.81±12.15	19.03±36.58	42.55±2.27	66.86±6.22	0.70±0.03	41.31±2.68	0.53±0.94	55.63±2.19	67.79±5.68	50.78±2.95	39.30±8.67	11.70±22.12	42.99±1.80	63.21±5.05	0.68±0.05
test ktn SAM											31.23±8.82	3.89±7.36	46.26±6.13	43.54±13.09	37.99±8.41	31.01±14.63	16.25±26.67	36.03±5.23	40.74±12.15	0.54±0.10
test WT floral											43.76±1.21	0.84±0.81	56.60±1.64	73.84±2.22	52.97±1.31	45.82±11.24	21.75±31.29	42.98±1.39	72.75±5.69	0.72±0.01
test ktn floral											44.91±0.89	1.81±2.44	58.31±0.92	74.59±1.65	54.34±0.84	48.19±11.24	28.44±31.59	44.79±0.80	71.34±3.73	0.72±0.01

topoAndBio

	train F1	train c0 F1	train c1 F1	train c2 F1	train Acc	train precision	train c0 precision	train c1 precision	train c2 precision	train Auc	val F1	val c0 F1	val c1 F1	val c2 F1	val Acc	val precision	val c0 precision	val c1 precision	val c2 precision	val Auc
split 0	73.10	72.18	72.24	74.88	73.09	73.12	71.54	72.28	75.52	0.88	64.07	60.45	64.05	67.70	64.04	64.12	60.01	63.32	69.05	0.81
split 1	70.33	68.57	68.90	73.51	70.33	70.33	68.92	68.57	73.51	0.86	70.92	72.49	71.55	68.72	70.92	70.94	73.29	70.21	69.32	0.88
split 2	71.64	70.53	71.79	72.61	71.64	71.65	70.71	71.00	73.26	0.87	66.07	64.47	64.84	68.90	66.03	66.16	63.48	64.19	70.80	0.83
split 3	71.08	69.23	70.05	73.97	71.08	71.10	69.98	69.18	74.13	0.86	70.03	70.84	70.16	69.08	70.06	70.35	66.38	71.90	72.78	0.87
split 4	69.63	67.89	68.71	72.30	69.64	69.64	68.55	68.05	72.32	0.86	71.57	68.37	70.73	75.61	71.64	71.69	72.15	68.58	74.34	0.88
split 5	71.27	69.64	70.44	73.74	71.27	71.27	69.72	70.45	73.64	0.87	69.30	66.82	68.73	72.34	69.37	69.38	69.86	68.71	69.57	0.85
train mean	71.18±1.08	69.67±1.39	70.35±1.32	73.50±0.86	71.18±1.08	71.18±1.09	69.90±1.02	69.92±1.47	73.73±0.97	0.87±0.01	68.66±2.70	67.24±3.99	68.34±2.89	70.39±2.74	68.68±2.73	68.77±2.72	67.53±4.73	67.82±3.09	70.98±1.96	0.85±0.03
test WT SAM	72.54±5.52	71.32±6.24	72.21±7.67	74.09±5.49	72.60±5.51	72.82±5.60	71.38±5.93	71.69±7.03	75.40±7.37	0.88±0.04	66.27±4.24	67.78±2.89	65.44±4.85	65.59±9.55	66.62±3.64	66.95±3.83	65.39±4.50	67.43±6.42	68.03±4.36	0.85±0.02
test ktn SAM											42.47±6.30	43.38±6.16	45.09±4.58	38.95±9.27	42.57±6.15	43.18±7.22	41.31±5.74	44.29±4.02	43.95±14.43	0.60±0.07
test WT floral											62.17±6.83	58.63±9.24	53.91±12.13	73.98±0.83	62.70±6.32	62.56±6.91	57.54±9.41	58.30±10.75	71.85±3.41	0.80±0.06
test ktn floral											62.58±4.80	59.63±5.81	55.54±7.98	72.59±1.87	62.99±4.76	63.30±5.23	58.47±8.22	62.04±8.79	69.39±3.33	0.81±0.03

r < 0.3

	train F1	train c0 F1	train c1 F1	train c2 F1	train Acc	train precision	train c0 precision	train c1 precision	train c2 precision	train Auc	val F1	val c0 F1	val c1 F1	val c2 F1	val Acc	val precision	val c0 precision	val c1 precision	val c2 precision	val Auc
split 0	60.57	63.27	66.33	52.09	60.89	60.60	60.84	64.15	56.82	0.78	52.40	53.12	59.99	44.09	52.47	52.51	56.24	57.63	43.66	0.71
split 1	58.77	61.96	65.25	49.10	59.18	58.76	60.32	62.16	53.81	0.77	62.15	66.48	68.87	51.11	62.34	62.04	65.98	67.24	52.89	0.81
split 2	61.53	65.68	68.34	50.58	62.01	61.52	63.39	65.26	55.91	0.80	54.39	57.04	58.62	47.51	54.58	54.59	52.97	59.91	50.89	0.73
split 3	57.96	61.76	64.93	47.19	58.45	57.94	59.53	61.92	52.36	0.77	60.73	66.52	69.41	46.25	61.66	61.23	59.46	69.21	55.02	0.80
split 4	59.22	62.70	65.73	49.23	59.67	59.24	60.48	62.69	54.57	0.77	61.13	64.14	69.72	49.53	62.33	62.68	64.60	60.35	63.08	0.79
split 5	58.96	62.81	65.64	48.43	59.42	58.94	61.06	62.33	53.43	0.77	57.65	56.86	65.86	50.21	57.69	57.80	60.47	63.80	49.11	0.76
train mean	59.50±1.19	63.03±1.29	66.04±1.12	49.44±1.56	59.94±1.18	59.50±1.20	60.94±1.20	63.08±1.21	54.48±1.51	0.78±0.01	58.07±3.63	60.69±5.24	65.41±4.51	48.12±2.43	58.51±3.91	58.47±3.85	59.95±4.49	63.02±4.14	52.44±5.93	0.77±0.04
test WT SAM	59.68±5.40	64.43±6.73	66.57±6.76	48.03±7.95	60.53±5.54	60.56±6.18	62.37±6.77	63.36±6.84	55.95±9.82	0.79±0.05	54.96±4.72	61.71±6.11	58.95±8.06	44.22±7.43	55.96±4.47	56.17±5.13	57.50±4.27	61.53±8.26	49.47±8.35	0.76±0.03
test ktn SAM											44.27±1.38	47.57±5.27	42.66±4.56	42.57±6.60	44.74±1.46	45.00±0.68	44.70±5.37	43.27±1.54	47.03±4.78	0.62±0.02
test WT floral											46.94±9.34	51.15±6.89	42.91±18.20	46.76±5.93	47.89±8.06	47.87±9.29	49.36±9.05	46.61±17.00	47.66±3.86	0.66±0.10
test ktn floral											46.50±5.43	51.12±7.21	43.59±10.89	44.77±5.59	47.50±4.86	48.38±5.13	49.63±7.87	51.17±9.62	44.34±2.34	0.66±0.05

unweighted

	train F1	train c0 F1	train c1 F1	train c2 F1	train Acc	train precision	train c0 precision	train c1 precision	train c2 precision	train Auc	val F1	val c0 F1	val c1 F1	val c2 F1	val Acc	val precision	val c0 precision	val c1 precision	val c2 precision	val Auc
split 0	57.50	62.75	64.30	45.43	58.19	57.57	59.52	60.99	52.21	0.76	51.22	54.82	57.49	41.37	51.70	51.26	54.83	53.18	45.78	0.70
split 1	55.18	60.08	61.94	43.53	55.85	55.24	57.73	58.09	49.90	0.74	61.80	69.88	67.79	47.72	62.50	61.77	68.15	63.32	53.84	0.80
split 2	56.87	63.94	63.57	43.10	57.78	56.97	60.39	59.69	50.83	0.76	52.97	56.53	57.81	44.58	53.30	53.01	53.41	56.81	48.82	0.72
split 3	55.91	60.29	62.33	45.10	56.45	55.93	58.16	58.90	50.73	0.75	54.60	64.25	66.10	33.46	56.83	54.90	58.21	60.82	45.66	0.77
split 4	55.43	60.58	62.21	43.51	56.18	55.57	58.19	57.90	50.64	0.74	57.12	62.12	66.70	42.53	58.32	57.64	57.06	62.56	53.29	0.78
split 5	55.40	60.82	62.40	42.97	56.20	55.53	58.13	58.15	50.32	0.75	56.10	62.77	62.87	42.66	56.99	56.26	60.48	57.92	50.38	0.76
train mean	56.05±0.85	61.41±1.43	62.79±0.85	43.94±0.97	56.77±0.88	56.14±0.84	58.69±0.94	58.95±1.09	50.77±0.72	0.75±0.01	55.64±3.37	61.73±4.98	63.13±4.15	42.05±4.35	56.61±3.49	55.81±3.38	58.69±4.80	59.10±3.52	49.63±3.24	0.75±0.04
test WT SAM	55.72±5.03	62.24±7.94	63.63±7.76	41.29±7.27	57.13±5.58	56.70±5.38	59.00±6.68	58.67±6.91	52.44±8.01	0.76±0.06	55.44±4.86	62.80±6.12	64.01±5.59	39.52±10.60	57.08±5.02	56.58±5.58	59.56±4.18	59.03±5.86	51.16±10.51	0.77±0.04
test ktn SAM											41.23±4.11	46.55±4.59	44.43±3.81	32.70±7.63	42.49±3.93	44.14±4.22	42.92±4.59	40.03±3.59	49.47±6.31	0.60±0.03
test WT floral											48.89±7.33	50.89±8.20	49.38±10.92	46.41±5.60	49.06±7.33	49.10±7.18	49.24±9.00	49.66±8.94	48.41±6.13	0.67±0.08
test ktn floral											49.54±4.33	51.99±6.35	51.05±6.97	45.58±2.41	49.70±4.43	49.66±4.33	50.95±6.14	50.98±6.39	47.05±1.92	0.68±0.05

Development • Supplementary information

References

Alim, K., Hamant, O., Boudaoud, A., 2012. Regulatory role of cell division rules on tissue growth heterogeneity. Frontiers in plant science 3, 174.
Barbier de Reuille, P., Routier-Kierzkowska, A.-L., Kierzkowski, D., Bassel, G.W., Schüpbach, T., Tauriello, G., Bajpai, N., Strauss, S., Weber, A., Kiss, A., Burian, A., Hofhuis, H., Sapala, A., Lipowczan, M., Heimlicher, M.B., Robinson, S., Bayer, E.M., Basler, K., Koumoutsakos, P., Roeder, A.H.K., Aegerter-Wilmsen, T., Nakayama, N., Tsiantis, M., Hay, A., Kwiatkowska, D., Xenarios, I., Kuhlemeier, C., Smith, R.S., 2015. MorphoGraphX: A platform for quantifying morphogenesis in 4D. eLife 4, 5864.
Beauzamy, L., Louveaux, M., Hamant, O., Boudaoud, A., 2015. Mechanically, the Shoot Apical Meristem of Arabidopsis Behaves like a Shell Inflated by a Pressure of About 1 MPa. Frontiers in plant science 6, 1038.
Besson, S., Dumais, J., 2011. Universal rule for the symmetric division of plant cells. Proceedings of the National Academy of Sciences of the United States of America 108, 6294–6299.
Bhavsar, H., Panchal, M.H., 2012. A review on support vector machine for data classification. International Journal of Advanced Research in Computer Engineering & Technology (IJARCET) 1, 185–189.
Breuer, D., Nowak, J., Ivakov, A., Somssich, M., Persson, S., Nikoloski, Z., 2017. System-wide organization of actin cytoskeleton determines organelle transport in hypocotyl plant cells. Proceedings of the National Academy of Sciences of the United States of America 114, E5741-E5749.
Camacho, D.M., Collins, K.M., Powers, R.K., Costello, J.C., Collins, J.J., 2018. Next-Generation Machine Learning for Biological Networks. Cell 173, 1581–1592.
D'Ario, M., Sablowski, R., 2019. Cell Size Control in Plants. Annual review of genetics 53, 45–65.
D'Ario, M., Tavares, R., Schiessl, K., Desvoyes, B., Gutierrez, C., Howard, M., Sablowski, R., 2021. Cell size controlled in plants using DNA content as an internal scale. Science (New York, N.Y.) 372, 1176–1181.
Dewitte, W., Murray, J.A., 2003. The Plant Cell Cycle. Annual Review of Plant Biology 54, 235–264.
Eng, R.C., Schneider, R., Matz, T.W., Carter, R., Ehrhardt, D.W., Jönsson, H., Nikoloski, Z., Sampathkumar, A., 2021. KATANIN and CLASP function at different spatial scales to mediate microtubule response to mechanical stress in Arabidopsis cotyledons. Current biology : CB. https://doi.org/10.1016/j.cub.2021.05.019.
Gibson, W.T., Veldhuis, J.H., Rubinstein, B., Cartwright, H.N., Perrimon, N., Brodland, G.W., Nagpal, R., Gibson, M.C., 2011. Control of the mitotic cleavage plane by local epithelial topology. Cell 144, 427–438.
Hartig, K., Beck, E., 2006. Crosstalk between auxin, cytokinins, and sugars in the plant cell cycle. Plant biology (Stuttgart, Germany) 8, 389–396.
Jackson, M.D.B., Duran-Nebreda, S., Kierzkowski, D., Strauss, S., Xu, H., Landrein, B., Hamant, O., Smith, R.S., Johnston, I.G., Bassel, G.W., 2019. Global Topological Order Emerges through Local Mechanical Control of Cell Divisions in the Arabidopsis Shoot Apical Meristem. Cell systems 8, 53-65.e3.
Jones, A.R., Forero-Vargas, M., Withers, S.P., Smith, R.S., Traas, J., Dewitte, W., Murray, J.A.H., 2017. Cell-size dependent progression of the cell cycle creates homeostasis and flexibility of plant cell size. Nature communications 8, 15060.
Katz, L., 1953. A new status index derived from sociometric analysis. Psychometrika 18, 39–43.
Kitagawa, M., Jackson, D., 2017. Plasmodesmata-Mediated Cell-to-Cell Communication in the Shoot Apical Meristem: How Stem Cells Talk. Plants (Basel, Switzerland) 6.
Komis, G., Luptovčiak, I., Ovečka, M., Samakovli, D., Šamajová, O., Šamaj, J., 2017. Katanin Effects on Dynamics of Cortical Microtubules and Mitotic Arrays in Arabidopsis thaliana Revealed by Advanced Live-Cell Imaging. Frontiers in plant science 8, 866.
Li, Y., Liu, D., López-Paz, C., Olson, B.J., Umen, J.G., 2016. A new class of cyclin dependent kinase in Chlamydomonas is required for coupling cell size to cell division. eLife 5, e10767.

Development • Supplementary information

Louveaux, M., Julien, J.-D., Mirabet, V., Boudaoud, A., Hamant, O., 2016. Cell division plane orientation based on tensile stress in Arabidopsis thaliana. Proceedings of the National Academy of Sciences of the United States of America 113, E4294-303.
Marchiori, M., Latora, V., 2000. Harmony in the small-world. Physica A: Statistical Mechanics and its Applications 285, 539–546.
Nowak, J., Eng, R.C., Matz, T., Waack, M., Persson, S., Sampathkumar, A., Nikoloski, Z., 2021. A network-based framework for shape analysis enables accurate characterization of leaf epidermal cells. Nature communications 12, 458.
Pisner, D.A., Schnyer, D.M., 2020. Support vector machine. In: Machine Learning. Elsevier, pp. 101–121.
Qi, F., Zhang, F., 2019. Cell Cycle Regulation in the Plant Response to Stress. Frontiers in plant science 10, 1765.
Reddy, G.V., Heisler, M.G., Ehrhardt, D.W., Meyerowitz, E.M., 2004. Real-time lineage analysis reveals oriented cell divisions associated with morphogenesis at the shoot apex of Arabidopsis thaliana. Development (Cambridge, England) 131, 4225–4237.
Sahlin, P., Jönsson, H., 2010. A modeling study on how cell division affects properties of epithelial tissues under isotropic growth. PloS one 5, e11750.
Sampathkumar, A., 2020. Mechanical feedback-loop regulation of morphogenesis in plants. Development (Cambridge, England) 147.
Sampathkumar, A., Krupinski, P., Wightman, R., Milani, P., Berquand, A., Boudaoud, A., Hamant, O., Jönsson, H., Meyerowitz, E.M., 2014. Subcellular and supracellular mechanical stress prescribes cytoskeleton behavior in Arabidopsis cotyledon pavement cells. eLife 3, e01967.
Schoof, H., Lenhard, M., Haecker, A., Mayer, K.F., Jürgens, G., Laux, T., 2000. The Stem Cell Population of Arabidopsis Shoot Meristems Is Maintained by a Regulatory Loop between the CLAVATA and WUSCHEL Genes. Cell 100, 635–644.
Shapiro, B.E., Tobin, C., Mjolsness, E., Meyerowitz, E.M., 2015. Analysis of cell division patterns in the Arabidopsis shoot apical meristem. Proceedings of the National Academy of Sciences 112, 4815–4820.
Shi, B., Guo, X., Wang, Y., Xiong, Y., Wang, J., Hayashi, K.-I., Lei, J., Zhang, L., Jiao, Y., 2018. Feedback from Lateral Organs Controls Shoot Apical Meristem Growth by Modulating Auxin Transport. Developmental cell 44, 204-216.e6.
Shimotohno, A., Aki, S.S., Takahashi, N., Umeda, M., 2021. Regulation of the Plant Cell Cycle in Response to Hormones and the Environment. Annual Review of Plant Biology 72, 273–296.
Uyttewaal, M., Burian, A., Alim, K., Landrein, B., Borowska-Wykręt, D., Dedieu, A., Peaucelle, A., Ludynia, M., Traas, J., Boudaoud, A., Kwiatkowska, D., Hamant, O., 2012. Mechanical stress acts via katanin to amplify differences in growth rate between adjacent cells in Arabidopsis. Cell 149, 439–451.
Varner, J.E., Lin, L.-S., 1989. Plant cell wall architecture. Cell 56, 231–239.
Veylder, L. de, Beeckman, T., Inzé, D., 2007. The ins and outs of the plant cell cycle. Nature reviews. Molecular cell biology 8, 655–665.
Wang, Y., Sampathkumar, A., 2020. Live Cell Imaging of Microtubule Cytoskeleton and Micromechanical Manipulation of the Arabidopsis Shoot Apical Meristem. Journal of visualized experiments : JoVE.
Wangenheim, D. von, Fangerau, J., Schmitz, A., Smith, R.S., Leitte, H., Stelzer, E.H.K., Maizel, A., 2016. Rules and Self-Organizing Properties of Post-embryonic Plant Organ Cell Division Patterns. Current biology : CB 26, 439–449.
Willis, L., Refahi, Y., Wightman, R., Landrein, B., Teles, J., Huang, K.C., Meyerowitz, E.M., Jönsson, H., 2016. Cell size and growth regulation in the Arabidopsis thaliana apical stem cell niche. Proceedings of the National Academy of Sciences of the United States of America 113, E8238-E8246.
