# Supplementary material for: Topological properties accurately predict cell division events and organization of shoot apical meristem in Arabidopsis thaliana
Source: Development. 2022 Aug 16;149(16):dev201024. doi: 10.1242/dev.201024 (PMC9481972; doi:10.1242/dev.201024)
Supplement: Reviewer comments [file develop-149-201024_review_history.pdf]

Topological properties accurately predict cell division events and organization of shoot apical meristem in *Arabidopsis thaliana*

Timon Werner Matz, Yang Wang, Ritkia Kulshreshtha, Arun Sampathkumar and Zoran Nikoloski
DOI: 10.1242/dev.201024

Editor: Yka Helariutta

Review timeline
Original submission: 5 October 2021
Editorial decision: 15 November 2021
Resubmission: 15 June 2022
Accepted: 15 July 2022

Original submission

First decision letter

MS ID#: DEVELOP/2021/200248

MS TITLE: Topological properties accurately predict cell division events and organization of Arabidopsis thaliana's shoot apical meristem

AUTHORS: Timon Werner Matz, Yang Wang, Ritika Kulshreshtha, Arun Sampathkumar, and Zoran Nikoloski
ARTICLE TYPE: Research Article

Dear Dr. Nikoloski,

I have now received all the referees reports on the above manuscript, and have reached a decision. The referees' comments are appended below, or you can access them online: please go to BenchPress and click on the 'Manuscripts with Decisions' queue in the Author Area.

As you will see from their reports, the referees recognise the potential of your work, but they also raise significant concerns about it. Given the nature of these concerns, I am afraid I have little choice other than to reject the paper at this stage.

However, having evaluated the paper, I do recognise the potential importance of this work. I would therefore be prepared to consider as a new submission an extension of this study that contains new experiments, data and discussions and that address fully the major concerns of the referees. The work required goes beyond a standard revision of the paper. Please bear in mind that the referees (who may be different from the present reviewers) will assess the novelty of your work in the context of all previous publications, including those published between now and the time of resubmission.

© 2022. Published by The Company of Biologists under the terms of the Creative Commons Attribution License (https://creativecommons.org/licenses/by/4.0/).

Yours sincerely,

Yka Helariutta
Handling Editor
Development

Reviewer 1

Advance Summary and Potential Significance to Field

The MSC titled "Topological properties accurately predict cell division events and organization of Arabidopsis thaliana's shoot apical meristem" by Matz et al., submitted to Development (DEVELOP-2021-200248v1-Nikoloski), presents a computational approach to study the regulation of cell divisions in the shoot apical meristem, i.e. the conditions on which the cell progress to division takes place, and factors that regulate the orientation of division plane. This approach is based on support vector machines where classifiers are "trained" on topological parameters obtained from network representation of cellular pattern of the SAM surface, as well as cell and wall size parameters. The computations show that an individual cell and cell wall size combined with its topological traits are decisive for the cell division occurrence and its topology changes due to this division.
The strength of this analysis is in the large set of data used for the modeling and comprehensive set of topological parameters being employed. Moreover, the problem of cell division regulation in plant meristems is fundamental for our understanding of plant development while this MSC provides some new arguments on the role of different cell and tissue scale factors in this regulation.

Comments for the author

In my opinion the MSC has some flaws that need to be dealt with by the Authors. First, the Results and Methods are hard to follow because of insufficient explanation provided (please see specific comments below) and usage of jargon-like terms (e.g. line 55 - "cell display higher centralities"; l.132 - "to ensure balancing of cell labels"). My background is biology, as that of numerous readers of Development journal. Nevertheless, although I am using and developing some computation and modeling approach in my research, I really had problems to follow the Results text. Second, the Discussion in my opinion has not enough reference to the topological parameters examined and their putative biological interpretation. Moreover, interpreting the data obtained for katanin mutant would provide arguments in favor of the presented method. Specific comments are listed below in order of their appearance in the text.

Major comments:

1. In Introduction it may help the reader if general description of a network representation and classes of topological parameters used is provided.
2. In Results, where the different topological parameters are referred to, or in legend of Supplement Table 1, it would really help if some brief explanation were given, like that for betweenness in the main text. Moreover, a legend of the symbols used in Suppl Table 1 is missing. Also explaining the difference in meaning of training and validation accuracies would be welcomed.
3. In my opinion, more attention need to be paid to the obtained data in the Discussion. I miss more explanation on what would be the biomechanical significance of various topological and "biological" features considered in the model. The Authors may also describe in more detail the extent to which the topological parameters represent a tissue scale. Am I right that most of them refer to the cell and its immediate neighbors? Are the

© 2022. Published by The Company of Biologists under the terms of the Creative Commons Attribution License (https://creativecommons.org/licenses/by/4.0/).

topological features indirectly representing the cell shape? Adding such comments to the text would make it more appealing to biologists.

Minor comments:
Lines 23/4 -what is meant by "on par"?

l. 48-51 - this sentence is not clear. Please, explain what is meant by "increase in size" here. Is it growth? And what is the constant increase in the adder model? For sure these are explained in the cited paper, but the reader may be not familiar with these details.
l.73 - "analysis of IMAGED phenotypes"?

l.97 - inflorescence SAM?

l.100-101 and corresponding Methods section - the description of SAM center identification is not clear. Which point is the highest depends on how the apex is oriented (not so easy to find the right orientation when primordia are spirally arranged), while the highest curvature rule is not convincing (what if the SAM were slightly flattened at the center?).
l.109-10 - cell surface area, or rather the surface area of outer periclinal cell wall is mentioned as a biological feature here, but there are more of them later on, also in Results (e.g. l. 178-9). Please, make it uniform.
l.115 - please consider explaining why this weight is used here

l.251 - ADJACENT ?

l.293 - do the Authors refer to mechanical protection and cell proliferation functions ? It is not clear.
l.298-9 - a contrast to Jackson et al. 2019 paper is pointed here. Providing more arguments, or elaboration of this discrepancy is needed in my opinion.
l.370-1 - is it really a "soil thickness" that is referred to?

l.391 - extractED

l.410-11 - the text in parentheses is not clear

l. 494-6 - the style is strange, please rephrase

l.534 - I am not able to interpret the line widths ...

figure 2A - what are the "TP & TN, FN & FP" ?

suppl figure 5 - "testing - ktn" is only in color code explanation and not in bars

Suppl table 1 - year for Estrada citation is not complete

Legends in general are quite repetitive, e.g. in classifiers explanation. It would be better to use this space to explain the model & terms in more detail.

Reviewer 2

Advance Summary and Potential Significance to Field

Matz et al. collected time-lapse images of Arabidopsis shoot meristems and made extensive use of machine learning to identify topological features that best predict cell division. They argue that topological features are more accurate predictors of cell division

© 2022. Published by The Company of Biologists under the terms of the Creative Commons Attribution License (https://creativecommons.org/licenses/by/4.0/).

that cell size alone, and that local topology is useful to predict subsequent changes in tissue topology.

The question of how cell geometry, connectivity to neighbors and cell mechanics could affect cell division parameters is important and the methods used in this paper are cutting edge. However, I have concerns about conceptual advance and technical issues, as detailed below.

Comments for the author

1. I am not convinced that this work is a major advance. At the end of the introduction, the authors say: "Although there are attempts of combining network properties with imaging data from SAMs, minor progress has been achieved in predicting individual cells divisions in this plant tissue". Jackson 2019 had already made important progress in analyzing cell topology in the shoot meristem cells, linking it to cell division orientation and discussing the biological relevance of topological parameters. Jackson et al. 2019 also covered specific aspects of the paper by Matz et al., such as the importance of betweenness centrality and the analysis of cell topology in the katanin mutant. Matz et al. now show that a combination of topological features has a slightly higher predictive power of cell division timing compared to cell area alone, and that topological features of cells that do not divide within 24 h can predict subsequent tissue topology. In my view, the conceptual advance is relatively minor.

2. The topological features that improve the ability to predict cell division are not really independent of cell size. Although an attempt was made to focus on those with the weakest correlation with size, a Pearson correlation value of 0.5-0.7 does not show independence. Furthermore, each of the topological features might correlate imperfectly with size for different reasons, so the combined features would be expected to better capture the effects of cell size. Perhaps the slightly improved predictive power of topological features results from a better approximation of cell volume, which is more relevant than the cell area measurements used by the authors. Mechanical constraints from neighboring cells, which would reflect topological features, might affect how much a cell's thickness diverges from the average thickness of the L1 layer.

3. An important point related to 3 above is that due to limitations in their image analysis methods, the authors focused exclusively on the L1 layer. To explore ideas on the effect of cell topology and tissue mechanics, it would be necessary to include the inner cell layers. I do not consider this simply an interesting future direction, as presented in the Discussion, but as essential to properly interpret the biological consequences of cell and tissue topology.

4. Training sets were based on 4 meristems, but independent testing of predictions relied only on one meristem per genotype. Considering that overall topology is a property of individual meristems, this means that testing relied on a single biological replicate. I think that biological replication needs to be increased.

5. I would have liked to see clearer hypotheses for how topological features could predict cell division independently of size - the authors allude to differences in cell communication paths and mechanical effects, but could they associate these hypotheses with the topological features with the highest predictive power, and more importantly, how could they test that the topological features influence the timing of cell division independently of cell size?

© 2022. Published by The Company of Biologists under the terms of the Creative Commons Attribution License (https://creativecommons.org/licenses/by/4.0/).

6. In the end, I am not convinced that the authors can exclude that topology and tissue mechanics primarily affect the placement of division planes, while cell size is the main determinant of the timing of cell division.

Reviewer 3

Advance Summary and Potential Significance to Field

Studying the reciprocal effects of cell behaviors and tissue growth and morphology is critical to understand development and maintenance of tissues and organs. In this manuscript, authors use live imaging data set of the L1 layers cells in Arabidopsis SAMs to develop a network representation method to predict cell divisions and their effects on shoot apical meristem topology. Authors with the use various topological properties besides cell size show that the cell division predictions in SAMs could be improved. The idea of using machine learning to predict cell division events is definitely a worthwhile endeavor, and the use of the so-called topological properties is sensible.

Comments for the author

Studying the reciprocal effects of cell behaviors and tissue growth and morphology is critical to understanding the development and maintenance of tissues and organs. In this manuscript, the authors use live imaging data set of the cells of the L1 layer in Arabidopsis SAMs to develop a network representation method to predict cell divisions and their effects on the shoot apical meristem topology. Authors with the use of various topological properties besides cell size show that the cell division predictions in SAMs could be improved. Finally, they use microtubules disrupted mutants to show that a supracellular network may play a role in coordinating individual cell behaviors with tissue morphology. The idea of using machine learning to predict cell division events is definitely a worthwhile endeavor, and the use of the so-called topological properties is sensible. However, there are some questions about how the method works that need to be clarified and the current analysis needs to be extended to other regions of SAMs. The Github link did not work, so it was not possible to see the code.

1. Authors focus on the central 30uM of the L1 layers of SAMs, this area does not truly represent drastically distinct topologies. Extending the current analysis of the lateral edges of the peripheral zone and especially cells of the organ boundary region will rigorously test the methodology in predicting cell divisions and moreover may provide new insights into the interplay between cell division and tissue morphology. Authors should be able to use the existing Z-stacks to carry out this analysis. If authors can use the adjacent floral bud imagery, that would definitely benefit the current analysis as dramatic morphological changes ensue during floral bud development.
2. The paper keeps referring to a "network". It is not clear what this network is. Given the use of deep neural networks in machine learning (ML), it gives an impression that they are related, but it does not seem to be the case. What the authors seem to refer to is a graph. Referring to it as a network is fine, but this needs to be clarified, probably with an image of how this is constructed. Fig. 1 gives a simple example, but details on how this graph is constructed at scale needs to be provided.
3. It is not clear how the different features used in the machine learning approach were computed. It seems to that these features, at least for the training side, were generated manually (based on authors statement "To select the cells for the downstream analysis, we first manually determined the cells closest to the center of the SAM surface, given by the highest curvature."). Then the classifiers were trained with these features. If that is indeed the case, does machine learning really help? Generating these features

© 2022. Published by The Company of Biologists under the terms of the Creative Commons Attribution License (https://creativecommons.org/licenses/by/4.0/).

manually at scale is extremely time-consuming, since ML methods require large amounts of training data.
4. How were the features generated during the test time? If these were also generated manually, then the utility of the method is very limited. The most time-consuming part is still manual. If these features are generated automatically, then how are the errors of that process affecting the classification? In other words, if the features mentioned are generated through some computational pipeline, there will be associated errors. How is the classifier robust to such errors? There needs to be an empirical analysis of this.
5. Related to 4, can the features be predicted automatically and then the classifier trained? Basically, the input is the raw image and the output is the cell division event. On the training side, the model is trained just with raw images without any manual intervention after the image collection process. If the authors are doing that, this method is valuable. If not, the method may not contribute significantly.
6. The authors have data from 5 plants, with 4 being in the training-validation set and 1 in the test set. How to ensure that the model is not being overfit to this data? Doing a five-fold cross-validation is good, but I wonder if there is enough variability in the data for the trained model to be useful broadly. Why not try to train with larger amounts of SAM data? Is this related to the need for manual processing as mentioned above? Overall, the method need clarification to understand its contribution. The level of manual supervision needed in the training stage is probably a serious bottleneck to the generalizability of the approach (point 4 and 5above). If manual supervision is not needed, why not train on larger data volumes to ensure generalizability?

Author response to reviewers' comments

Reviewer 1 Advance Summary and Potential Significance to Field:
The MSC titled „Topological properties accurately predict cell division events and organization of Arabidopsis thaliana's shoot apical meristem" by Matz et al., submitted to Development (DEVELOP-2021-200248v1-Nikoloski), presents a computational approach to study the regulation of cell divisions in the shoot apical meristem, i.e. the conditions on which the cell progress to division takes place, and factors that regulate the orientation of division plane. This approach is based on support vector machines where classifiers are "trained" on topological parameters obtained from network representation of cellular pattern of the SAM surface, as well as cell and wall size parameters. The computations show that an individual cell and cell wall size combined with its topological traits are decisive for the cell division occurrence and its topology changes due to this division.

The strength of this analysis is in the large set of data used for the modeling and comprehensive set of topological parameters being employed. Moreover, the problem of cell division regulation in plant meristems is fundamental for our understanding of plant development while this MSC provides some new arguments on the role of different cell and tissue scale factors in this regulation.

We would like to thank the reviewer for the succinct summary noting the strengths of our study.

Reviewer 1 Comments for the Author:
In my opinion the MSC has some flaws that need to be dealt with by the Authors. First, the Results and Methods are hard to follow because of insufficient explanation provided (please see specific comments below) and usage of jargon- like terms (e.g. line 55 – "cell display higher centralities"; l.132 – "to ensure balancing of cell labels"). My background is biology, as that of numerous readers of Development journal. Nevertheless, although I am using and developing some computation and modeling approach in my research, I really had problems to follow the Results text. Second, the Discussion in my opinion has not enough reference to the topological parameters examined and their putative biological interpretation. Moreover, interpreting the data obtained for katanin

© 2022. Published by The Company of Biologists under the terms of the Creative Commons Attribution License (https://creativecommons.org/licenses/by/4.0/).

mutant would provide arguments in favor of the presented method. Specific comments are listed below in order of their appearance in the text.

In the updated version of the manuscript, we streamlined the jargon (while paying attention to field-specific wording) and provide detailed explanations for what is performed in the present study. To this end, we provided a brief interpretation of the properties in the updated version of the manuscript. Importantly, we expand the discussion of the results pertaining to the katanin mutant.

Major comments:

1. In Introduction it may help the reader if general description of a network representation and classes of topological parameters used is provided.

We have added a section on network representation of imaging data, the meaning of nodes / edges in each of the mentioned representations and how we use them to generate the results presented in our manuscript. Moreover, we briefly introduce and discuss network properties that have been used to study imaging phenotypes, including cytoskeleton (Breuer et al. and Jackson et al.). [l. 77-84] We further included a section in the introduction explaining topological parameters. [l. 93-105]

2. In Results, where the different topological parameters are referred to, or in legend of Supplement Table 1, it would really help if some brief explanation were given, like that for betweenness in the main text. Moreover, a legend of the symbols used in Suppl Table 1 is missing. Also explaining the difference in meaning of training and validation accuracies would be welcomed.

All of these are clarified and resolved in the updated version of the manuscript. In addition to the explained topological properties in the introduction, we elaborate the most important topological properties of the division prediction classifier. [l. 188-201]

3. In my opinion, more attention need to be paid to the obtained data in the Discussion. I miss more explanation on what would be the biomechanical significance of various topological and “biological” features considered in the model. The Authors may also describe in more detail the extent to which the topological parameters represent a tissue scale. Am I right that most of them refer to the cell and its immediate neighbors? Are the topological features indirectly representing the cell shape? Adding such comments to the text would make it more appealing to biologists.

We would like to thank the reviewer for raising this point. Aligned with the two points above, we provide explanation for putative biomechanical significance of the properties / features used. In addition, we divide the network properties into local, local-global, and global to address the second part of the reviewer’s comment by pointing at their relation of the different types of network properties to tissue shape and tissue scale. [l. 95-104, 185-201]

Minor comments:
Lines 23/4 -what is meant by “on par”?

l. 48-51 – this sentence is not clear. Please, explain what is meant by “increase in size” here. Is it growth? And what is the constant increase in the adder model? For sure these are explained in the cited paper, but the reader may be not familiar with these details.

l.73 – “analysis of IMAGED phenotypes”?

l.97 – inflorescence SAM?

l.100-101 and corresponding Methods section – the description of SAM center identification is not clear. Which point is the highest depends on how the apex is oriented (not so easy to find the right orientation when primordia are spirally arranged), while the highest curvature rule is not convincing (what if the SAM were slightly flattened at the center?).

© 2022. Published by The Company of Biologists under the terms of the Creative Commons Attribution License (https://creativecommons.org/licenses/by/4.0/).

l.109-10 – cell surface area, or rather the surface area of outer periclinal cell wall is mentioned as a biological feature here, but there are more of them later on, also in Results (e.g. l. 178-9). Please, make it uniform.

l.115 – please consider explaining why this weight is used here

l.251 – ADJACENT ?

l.293 – do the Authors refer to mechanical protection and cell proliferation functions ? It is not clear.

l.298-9 – a contrast to Jackson et al. 2019 paper is pointed here. Providing more arguments, or elaboration of this discrepancy is needed in my opinion.

l.370-1 – is it really a “soil thickness” that is referred to?

l.391 – extractED

l.410-11 – the text in parentheses is not clear

l. 494-6 – the style is strange, please rephrase

l.534 – I am not able to interpret the line widths …

figure 2A – what are the “TP & TN, FN & FP” ?

suppl figure 5 – “testing – ktn” is only in color code explanation and not in bars

Suppl table 1 – year for Estrada citation is not complete

Legends in general are quite repetitive, e.g. in classifiers explanation. It would be better to use this space to explain the model & terms in more detail.

All of these are resolved by updating the respective lines. In addition, we streamlined the legends of the figures, while attempting to allow the figures to be understandable on their own.

Reviewer 2 Advance Summary and Potential Significance to Field:
Matz et al. collected time-lapse images of Arabidopsis shoot meristems and made extensive use of machine learning to identify topological features that best predict cell division. They argue that topological features are more accurate predictors of cell division that cell size alone, and that local topology is useful to predict subsequent changes in tissue topology.

The question of how cell geometry, connectivity to neighbors and cell mechanics could affect cell division parameters is important and the methods used in this paper are cutting edge. However, I have concerns about conceptual advance and technical issues, as detailed below.

We would like to thank the reviewer for recognizing the importance of the work. In our responses, below, we address all points raised by the reviewer regarding the advance that the study provides and the mentioned technical issues.

Reviewer 2 Comments for the Author:
1. I am not convinced that this work is a major advance. At the end of the introduction, the authors say: “Although there are attempts of combining network properties with imaging data from SAMs, minor progress has been achieved in predicting individual cells divisions in this plant tissue”. Jackson 2019 had already made important progress in analyzing cell topology in the shoot meristem cells, linking it to cell division orientation and discussing the biological relevance of topological parameters. Jackson et al. 2019 also covered specific aspects of the paper by Matz et al., such as the importance of betweenness centrality and the analysis of cell topology in the katanin mutant.

© 2022. Published by The Company of Biologists under the terms of the Creative Commons Attribution License (https://creativecommons.org/licenses/by/4.0/).

Matz et al. now show that a combination of topological features has a slightly higher predictive power of cell division timing compared to cell area alone, and that topological features of cells that do not divide within 24 h can predict subsequent tissue topology. In my view, the conceptual advance is relatively minor.

We are strongly convinced that this is a misreading of the work of Jackson et al. While the study of Jackson et al. shows an increase in area, volume, and three topological features between dividing and non-dividing cells, they fail to demonstrate that these features can predict cell division (even though the study claims to do so). For instance, in Jackson et al. the predictive power of area has a true positive rate of < 20%; in contrast, we can predict area with an f1- score of 72% for area. In addition, we can predict cell division with f1-score of 71% by using topological features that have no association to area (measured by Pearson correlations < 0.3) and consider it a conceptual advance since no machine learning model has relied on topological properties of this kind. Another conceptual novelty is given by the topological change upon division: While Jackson et al. suggest the placement of a new cell wall, we abstract the process and predict the changes caused by the dividing cell on the connectivity of their neighbours. Finally, our data are of higher quality and quantity: In Jackson et al. they claim to have 32 dividing cells used (over 3 layers in the first 11h time interval, see Jackson et al., Fig. 3) and investigating their summary files reveals that only 7 cells are dividing in the L1-layer. Even if they used their second time step dividing cells for the model creation, which is not evident, the actual number of dividing cells would be 122 with only 36 cells coming from the L1 layer, while in our study we compared 605 dividing cells (of L1-layer in 24h time interval), allowing us to train models that are of higher generalizability (due to the modelling strategy we have employed).

2. The topological features that improve the ability to predict cell division are not really independent of cell size. Although an attempt was made to focus on those with the weakest correlation with size, a Pearson correlation value of 0.5-0.7 does not show independence. Furthermore, each of the topological features might correlate imperfectly with size for different reasons, so the combined features would be expected to better capture the effects of cell size. Perhaps the slightly improved predictive power of topological features results from a better approximation of cell volume, which is more relevant than the cell area measurements used by the authors. Mechanical constraints from neighboring cells, which would reflect topological features, might affect how much a cell's thickness diverges from the average thickness of the L1 layer.

True independence of network properties from size is impossible as larger cells usually have more neighbours, but the use of local, local-global, and global-properties [explained in l 95-105] allow to overcome size dependency and allow us to capture information different from cell size. This holds equally well for 2D and 3D cell representations. This is due to the close relation between geometry and network representations thereof.

To convince the reviewer that our conclusions hold even when we use topological properties with even smaller association to area, we retrained models with the 17 features that show Pearson correlation < 0.3. While we are fully aware that Pearson correlation is not a measure of independence, we note that three of these 19 features are not significantly correlated to area. [l. 139-142] As detailed in the updated version of the manuscript, our findings show the same performance as using the full feature set. [l. 173-175]

3. An important point related to 3 above is that due to limitations in their image analysis methods, the authors focused exclusively on the L1 layer. To explore ideas on the effect of cell topology and tissue mechanics, it would be necessary to include the inner cell layers. I do not consider this simply an interesting future direction, as presented in the Discussion, but as essential to properly interpret the biological consequences of cell and tissue topology.

We have attempted to focus on the L1 layer as Jackson et al. already looked at pooled data and we are able to predict division events (see the answer regarding 2.). Thereby, we significantly improve on the study of Jackson et al. who have not done that.

4. Training sets were based on 4 meristems, but independent testing of predictions relied only on one meristem per genotype. Considering that overall topology is a property of individual meristems,

© 2022. Published by The Company of Biologists under the terms of the Creative Commons Attribution License (https://creativecommons.org/licenses/by/4.0/).

this means that testing relied on a single biological replicate. I think that biological replication needs to be increased.

The training set were based on 4 meristems with 12 time steps in total representing the biological replicates. We updated the text to better fit the notion of biological replicates being a time step per tissue. [l. 123-125, 152-156, 442- 444] Nevertheless, we have added three independent plants to demonstrate the predictive power of the model in unseen scenarios, training with 20 tissue time steps and predicting for 8 unseen tissue time steps now. [l. 471-475]

5. I would have liked to see clearer hypotheses for how topological features could predict cell division independently of size – the authors allude to differences in cell communication paths and mechanical effects, but could they associate these hypotheses with the topological features with the highest predictive power, and more importantly, how could they test that the topological features influence the timing of cell division independently of cell size?

We changed the svm kernel from rbf to linear [l.479, 528] (ensuring similar performance metrics) in order to allow for interpretation of the predictive power of features. [l. 185-201] The results are presented in the updated version of the manuscript.

6. In the end, I am not convinced that the authors can exclude that topology and tissue mechanics primarily affect the placement of division planes, while cell size is the main determinant of the timing of cell division.
See above for the extensive analyses. We would like to stress that the reviewer's argument holds for Jackson's study, which is revered by the reviewer. In addition, our study aims to offer an alternative model, rather than to exclude shown determinants.

Reviewer 3 Advance Summary and Potential Significance to Field:
Studying the reciprocal effects of cell behaviors and tissue growth and morphology is critical to understand development and maintenance of tissues and organs. In this manuscript, authors use live imaging data set of the L1 layers cells in Arabidopsis SAMs to develop a network representation method to predict cell divisions and their effects on shoot apical meristem topology. Authors with the use various topological properties besides cell size show that the cell division predictions in SAMs could be improved. The idea of using machine learning to predict cell division events is definitely a worthwhile endeavor, and the use of the so-called topological properties is sensible.

We would like to thank the reviewer for the positive evaluation and interest in the study!

Reviewer 3 Comments for the Author:
Studying the reciprocal effects of cell behaviors and tissue growth and morphology is critical to understanding the development and maintenance of tissues and organs. In this manuscript, the authors use live imaging data set of the cells of the L1 layer in Arabidopsis SAMs to develop a network representation method to predict cell divisions and their effects on the shoot apical meristem topology.
Authors with the use of various topological properties besides cell size show that the cell division predictions in SAMs could be improved. Finally, they use microtubules disrupted mutants to show that a supracellular network may play a role in coordinating individual cell behaviors with tissue morphology. The idea of using machine learning to predict cell division events is definitely a worthwhile endeavor, and the use of the so-called topological properties is sensible.
However, there are some questions about how the method works that need to be clarified and the current analysis needs to be extended to other regions of SAMs. The Github link did not work, so it was not possible to see the code.

The GitHub link was private, we have shared a password in the cover letter of the updated ms, and this will be made fully available upon publication.

1. Authors focus on the central 30uM of the L1 layers of SAMs, this area does not truly represent drastically distinct topologies. Extending the current analysis of the lateral edges of the peripheral zone and especially cells of the organ boundary region will rigorously test the methodology in predicting cell divisions and moreover may provide new insights into the interplay between cell

© 2022. Published by The Company of Biologists under the terms of the Creative Commons Attribution License (https://creativecommons.org/licenses/by/4.0/).

division and tissue morphology. Authors should be able to use the existing Z- stacks to carry out this analysis. If authors can use the adjacent floral bud imagery, that would definitely benefit the current analysis as dramatic morphological changes ensue during floral bud development.

In order to train any ML model, one needs use data obtained from different replicates with same techniques (to avoid confound effect) and test the generalizability of the model on unseen (so-called test) data. This allows us to examine the model's transferability to scenarios that were not previously encountered, both with respect to unseen data from same set-up (e.g. the SAM in different WT plants) or from different data distributions (e.g. the SAM from the katanin mutant, and its and WT floral meristems).

As the model assumes cells to be of the central region of the SAM and each cell to be at least two cells away from the boundary of the tissue/topology, using cell features from the peripheral region will result in artefacts. Alternatively, one could easily use the existing code/models and change the imaging setup to focus on the peripheral region.

To address the issue of transferability of the model, we tested the models on flower bud's central regions and found similar performance, demonstrating a rigorous test of the proposed methodology. [l. 208-215, 288-293]

2. The paper keeps referring to a "network". It is not clear what this network is. Given the use of deep neural networks in machine learning (ML), it gives an impression that they are related, but it does not seem to be the case. What the authors seem to refer to is a graph. Referring to it as a network is fine, but this needs to be clarified, probably with an image of how this is constructed. Fig. 1 gives a simple example, but details on how this graph is constructed at scale needs to be provided.

Per reviewers 1 & 3 request, we expand the introduction to explain what a network is and what it represents. This is already explained in Fig. 1B Graph = network (Borgatti and Halgin, 2011; Oh and Monge, 2016; Zhou et al., 2020). We explain how this is done from a practical perspective, e.g. using MorphographX and python with networkx, in the methods section. [l. 77-84, 93-105, 188-201]

3. It is not clear how the different features used in the machine learning approach were computed. It seems to that these features, at least for the training side, were generated manually (based on authors statement "To select the cells for the downstream analysis, we first manually determined the cells closest to the center of the SAM surface, given by the highest curvature."). Then the classifiers were trained with these features. If that is indeed the case, does machine learning really help? Generating these features manually at scale is extremely time-consuming, since ML methods require large amounts of training data.

All results are generated in a fully automated way, except for identifying the central cells and extracting the topology and geometric data from MGX. We clarified these aspects in the method section of the updated manuscript. [l. 446, 451]

4. How were the features generated during the test time? If these were also generated manually, then the utility of the method is very limited. The most time- consuming part is still manual. If these features are generated automatically, then how are the errors of that process affecting the classification? In other words, if the features mentioned are generated through some computational pipeline, there will be associated errors. How is the classifier robust to such errors? There needs to be an empirical analysis of this.

There are no errors from computational pipeline since deterministic algorithms are used to calculate these properties. Every tissue with the same organisation and geometric data of the cells results in the same topological and biological features.

5. Related to 4, can the features be predicted automatically and then the classifier trained? Basically, the input is the raw image and the output is the cell division event. On the training side, the model is trained just with raw images without any manual intervention after the image

© 2022. Published by The Company of Biologists under the terms of the Creative Commons Attribution License (https://creativecommons.org/licenses/by/4.0/).

collection process. If the authors are doing that, this method is valuable. If not, the method may not contribute significantly.

This is exactly what we did, see answer regarding point 4, above.

6. The authors have data from 5 plants, with 4 being in the training- validation set and 1 in the test set. How to ensure that the model is not being overfit to this data? Doing a five-fold cross-validation is good, but I wonder if there is enough variability in the data for the trained model to be useful broadly. Why not try to train with larger amounts of SAM data? Is this related to the need for manual processing as mentioned above? Overall, the method need clarification to understand its contribution. The level of manual supervision needed in the training stage is probably a serious bottleneck to the generalizability of the approach (point 4 and 5above). If manual supervision is not needed, why not train on larger data volumes to ensure generalizability?

We have learning curve, as a means for checking bias vs. variance [l. 181f, 271-273, 502-506] and we compare training with validation and test performance, where cells are classified based on unseen data, to double check for overfitting. The most similar model is trained by Jackson, et al. with 32 dividing cells over 3 layers (see legend of Fig.3) (with only 7 dividing cells from L1 layer, investigating the summary files) while we use 605 dividing cells from 28 WT SAM tissue time steps from L1 layer and other meristematic scenarios, including newly added plants. [l. 59-61, 471-475]
There is absolutely no manual supervision in this process - and this is stressed in the updated version of the manuscript. [l. 451]

References

Borgatti, S.P., Halgin, D.S., 2011. On Network Theory. Organization Science 22, 1168–1181.
Oh, P., Monge, P., 2016. Network Theory and Models. In: Bruhn Jensen, K., Craig, R.T. (Eds.) The international encyclopedia of communication theory and philosophy. R-Z. John Wiley & Sons, Chichester, UK, Hoboken, NJ, pp. 1-15.
Zhou, J., Cui, G., Hu, S., Zhang, Z., Yang, C., Liu, Z., Wang, L., Li, C., Sun, M., 2020. Graph neural networks: A review of methods and applications. AI Open 1, 57–81.

Resubmission

First decision letter

MS ID#: DEVELOP/2022/201024

MS TITLE: Topological properties accurately predict cell division events and organization of Arabidopsis thaliana's shoot apical meristem

AUTHORS: Timon Werner Matz, Yang Wang, Ritkia Kulshreshtha, Arun Sampathkumar, and Zoran Nikoloski
ARTICLE TYPE: Research Article

I am happy to tell you that your manuscript has been accepted for publication in Development, pending our standard ethics checks.

Reviewer 1

Advance summary and potential significance to field

As stated in my first review: the question of how cell geometry, connectivity to neighbors and cell mechanics could affect cell division parameters is important and the methods used in this paper are cutting edge.

© 2022. Published by The Company of Biologists under the terms of the Creative Commons Attribution License (https://creativecommons.org/licenses/by/4.0/).

However, I have concerns about the interpretation of the data and its limitations, as detailed below.

Comments for the author

The resubmitted version of Matz et al. is improved, especially in terms of biological replication and presentation for non-specialist readers. However, some of the issues I raised in my previous review have not been addressed. These issues particularly affect the biological interpretation of the results. Below are my comments on the new version, organised as much as possible around the points I raised in my first review.

Related to point 1:

I think the authors misunderstood my point. I mentioned the Jackson paper in detail not because I "revere" it (as in the reply to point 6), but because I object to claims of novelty that simply ignore identical or very similar claims that already exist in the literature. In the revised version, the authors discuss the limitations of the published work and discuss how their work goes further – that is the correct approach, and I think a similarly critical approach should be applied to their own analysis (see below).
Related to point 2:

As a compromise to facilitate image analysis, the authors used projected cell areas as a proxy for cell size.
The authors cannot be sure to what extent some of their topological features predict cell divisions independently of cell size, or whether these features capture information on cell size that has been sacrificed by adopting projected areas as the proxy. The author's response on this point did not quite hit the target:
predictions based on topological parameters with a low correlation with (projected) cell area still cannot resolve this issue. Incidentally, low values for Pearson's correlation coefficient can only exclude a linear relation; it could also be that these parameters are still dependent on cell size, but in a non-linear fashion.

Related to point 3:

Once again due to the choice of image processing method, the analysis is limited to the epidermal layer. The authors seem to imply that the role of topology can be analyzed in the L1 layer independently of the underlying cells. The L1 does have a predominant role when considering auxin transport, and to some extent for mechanical constraints. However, key biological processes that affect cell division and are expected to be influenced by cell topology, such as and access to nutrients and intercellular signals, need to be considered across the meristem layers. For this reason, I think the authors need to be clear about the limitations of interpreting data from the L1 alone. This issue was raised in my previous point 3, to which the authors replied that they "focus on the L1 layer as Jackson et al. already looked at pooled data and we are able to predict division events" – this seems to miss the biological implications of explaining L1 cell behavior as if it were independent of the underlying tissues.

Related to point 5:

I asked for clearer hypotheses for how topological features could predict cell division independently of size.
The author's reply focuses on SVM predictions: "We changed the svm kernel from rbf to linear [l.479, 528] (ensuring similar performance metrics". Perhaps this point has been missed because of a confusion between "prediction" based on a biological hypothesis (the point I tried to raise) and "prediction" as an output of machine learning.

Further issues:

a) In Figure 2A: the bulk of incorrect predictions seem to be failures to predict divisions, whilst most correct predictions appear to be for cells that did not divide. This gives the impression that

© 2022. Published by The Company of Biologists under the terms of the Creative Commons Attribution License (https://creativecommons.org/licenses/by/4.0/).

the SVMs perform poorly in predicting actual divisions and that most of the accuracy scores result from predicting non-dividing cells – is this a wrong impression given by the specific example in Figure 2A? To clarify this, the authors should provide separately the frequency of correctly predicted divisions, and correctly predicted non-divisions.

b) Still related to the independence of topological parameters and cell size: on page 6, lines 181-183, if SVMs based on projected area and on topology predicted divisions independently, then should not the combined criteria improve the performance (i.e., considering the approx. 25% of divisions not predicted by area alone, 75% of those should still be predicted by topology alone, giving an overall accuracy of nearly 94% if both SVMs were truly independent). To me the results suggest that both SVMs predict divisions using a largely overlapping pool of information present in the images.

c) Page 12. Line 381: "we showed that topological features alone sufficed to accurately predict local topological changes" – in contrast, Fig.3 seems to show that accurate prediction of local topology after division required combined information on both topology and cell size.

Conclusion, related to point 6:

Given all the points above, I remain unconvinced that the authors can exclude that topology and tissue mechanics primarily affect the placement of division planes, whilst cell size is the main determinant of the timing of cell division. The author's reply focuses on the merit of their work in comparison to Jackson (2019), but the key issues are wider than that.

Reviewer 2

Advance summary and potential significance to field

The MSC presents a computational approach to study the regulation of cell divisions in the shoot apical meristem, i.e. the conditions on which the cell progress to division takes place, and factors that regulate the orientation of division plane. The latter is not assessed as such but rather represented by the change in local cell topology. This approach is based on support vector machines where classifiers are "trained" on topological parameters obtained from network representation of cellular pattern of the SAM surface, as well as cell and wall size parameters. The computations show that an individual cell and cell wall size combined with its topological traits are decisive for the cell division occurrence and its topology changes due to this division.
The strength of this analysis is in the large set of data used for the modeling which in fact has been increased during the revision, and comprehensive set of topological parameters being employed. Moreover, the problem of cell division regulation in plant meristems is fundamental for our understanding of plant development while this MSC provides some new arguments on the role of different cell and tissue scale factors in this regulation.

Comments for the author

The MSC titled „Topological properties accurately predict cell division events and organization of Arabidopsis thaliana's shoot apical meristem" by Matz et al. has been resubmitted to Development after comprehensive revision according to the reviewers' suggestions. I feel entitled to make this comment being the reviewer #1 in the first round of evaluation.
In my opinion the MSC has been significantly improved during the revision, and only a few minor items need to be addressed by the Authors. Again, I have to admit that my background is biology. Nevertheless, it was now much easier for me to follow the Results text. Moreover, the Discussion now deals more with a putative biological interpretation of the topological parameters examined, and tries to interpret the data obtained for the katanin mutant. Below I list some specific minor comments in order of their appearance in the text.
1. Some acronyms are introduced in the text, like SAM, not at the first appearance of the full name in the text, or the introduction is more than once (e.g. SAM in lines 37 and 50).
2. line 129 – the usage of "central cell" here suggests that the Authors refer to the SAM center or central zone, while this is rather the central cell of the local network. Please rephrase.

© 2022. Published by The Company of Biologists under the terms of the Creative Commons Attribution License (https://creativecommons.org/licenses/by/4.0/).

3. line 132 – please explain here what exactly is meant by the shared cell wall (its length in SAM surface or rather its surface area?). I know it is not significant but just to be more precise.
4. lines 189-190 – Could the Authors explain here how the value of harmonic centrality is related to the cell and its neighbors' size? In general, it would help if somewhere here in the text, the centrality concept of graphs were briefly introduced.
5. line 192 – a brief explanation on the difference between walks and paths would be welcomed here.
6. line 233 – what "changes" are referred to here? I expect these are changes in topology rather than divisions …
7. lines 321, 406 – these phrases are really unclear. Please reword.
8. line 411 – the work by Gibson referred to here is on animal cells which have no cell walls and are not "glued" together. I suggest it is distinguished from works on plant cells.
9. Panel (D) is referred to in the legend of Figure 2, but there is no such panel in the figure.
10. In the Supplementary Table 1 numerous parameters are defined by equations. I could not find the explanation of symbols used in these equs?

© 2022. Published by The Company of Biologists under the terms of the Creative Commons Attribution License (https://creativecommons.org/licenses/by/4.0/).
